# A culture-independent approach, supervised machine learning, and the characterization of the microbial community composition of coastal areas across the Bay of Bengal and the Arabian Sea

**DOI:** 10.1186/s12866-024-03295-4

**Published:** 2024-05-10

**Authors:** Bhagwan Narayan Rekadwad, Yogesh Shreepad Shouche, Kamlesh Jangid

**Affiliations:** 1 National Centre for Microbial Resource, DBT - National Centre for Cell Science (DBT-NCCS), NCCS-Complex, Savitribai Phule Pune University (SPPU) Campus, Ganeshkhind Road, Pune, Maharashtra 411007 India; 2 MicrobeAI Lab, Division of Microbiology and Biotechnology, Yenepoya Research Centre, Yenepoya (Deemed to be University), University Road, Deralakatte, Mangalore, Karnataka 575018 India; 3 Bioenergy Group, DST-Agharkar Research Institute, Gopal Ganesh Agarkar Road, Pune, Maharashtra 411 004 India; 4 Gut Microbiology Research Division, SKAN Research Trust, Bangalore, Karnataka 560034 India

**Keywords:** Amplicon sequencing, Microbial composition and function, Coastal marine microbiome, Metagenomics, Operational taxonomic unit, Supervised machine learning

## Abstract

**Background:**

Coastal areas are subject to various anthropogenic and natural influences. In this study, we investigated and compared the characteristics of two coastal regions, Andhra Pradesh (AP) and Goa (GA), focusing on pollution, anthropogenic activities, and recreational impacts. We explored three main factors influencing the differences between these coastlines: The Bay of Bengal’s shallower depth and lower salinity; upwelling phenomena due to the thermocline in the Arabian Sea; and high tides that can cause strong currents that transport pollutants and debris.

**Results:**

The microbial diversity in GA was significantly higher than that in AP, which might be attributed to differences in temperature, soil type, and vegetation cover. 16S rRNA amplicon sequencing and bioinformatics analysis indicated the presence of diverse microbial phyla, including candidate phyla radiation (CPR). Statistical analysis, random forest regression, and supervised machine learning models classification confirm the diversity of the microbiome accurately. Furthermore, we have identified 450 cultures of heterotrophic, biotechnologically important bacteria. Some strains were identified as novel taxa based on 16S rRNA gene sequencing, showing promising potential for further study.

**Conclusion:**

Thus, our study provides valuable insights into the microbial diversity and pollution levels of coastal areas in AP and GA. These findings contribute to a better understanding of the impact of anthropogenic activities and climate variations on biology of coastal ecosystems and biodiversity.

**Supplementary Information:**

The online version contains supplementary material available at 10.1186/s12866-024-03295-4.

## Background

Coastal marine habitats are critical to the health of the environment and the economy as a whole. These include things such as providing habitat, recycling nutrients, protecting seashores, and safeguarding potential fishing zones [1]. Similarly, forest cover is crucial as a carbon sink, enabling carbon rhizodeposition back into the environment [2]. These ecosystems provide a variety of ecological, economic, and social benefits, such as habitat and biodiversity, climate regulation, coastal protection, food production, recreational activities, and a significant source of income for local economies through tourism [3, 4]. The health and services provided by these ecosystems are intrinsically tied to the microorganisms that reside in them, for example, pollution cleanup, disease, and drug discovery [5, 6]. The coastal marine microbiome is always active in critical habitats, flora, and fauna, such as corals, sponges, macroalgae, seagrasses, mangroves, and saltmarshes. These species are responsible for the stability of ecosystems [7]. Furthermore, because the health of coastal marine ecosystems is dependent on these creatures that create habitat, scientists have recognized the need to study macroorganisms and their microbiomes as a unified biological unit [8, 9]. As a result, substantial studies have been conducted in recent years on how microbiomes affect the phenotypic, physiologic, and developmental characteristics of the host [10–12]. Although we now have a better understanding of several fundamental concepts in coastal marine microbial ecology, coastal microbiome research is still in its early phases, particularly with regard to holobionts. This is especially true when contrasted to other domains of microbiome research, such as the human microbiome [13]. There are numerous unsolved questions at the time, making it difficult to establish how microbial processes affect the ecology of these habitats, both now and when the environment evolves in the future. Therefore, it is clear that we need to set priorities and come up with important questions for future research that will help us determine how microbial processes truly affect the biosphere and the health of coastal marine ecosystems [7, 14, 15].

A coastal-marine environment possesses a unique microbial community composition under particular environmental conditions. It tends to change with the fluctuating concentration of elements but remains steady with the physical and chemical nature of matter [16, 17]. The decrypted microbial diversity of the soil would act as a representative microbial pool for the entire region [18]. Marine microorganisms play a pivotal role in the ecosystem’s biogeochemical cycle [19]. These cycles involve the movement of nutrients and other substances between the ocean and the atmosphere as well as the movement of these substances within the ocean itself [20]. One important way that marine microorganisms contribute to biogeochemical cycles is through the process of photosynthesis. Many types of marine microorganisms, such as algae and cyanobacteria, are able to capture energy from sunlight and use it to convert carbon dioxide into organic matter through primary production, which supplies nutrition to microorganisms [21, 22]. Marine microorganisms also play a role in carbon sequestration and regulate carbon sinks [23]. In addition to these roles, marine microorganisms are also involved in the cycling of other important nutrients, such as nitrogen and phosphorus, which are essential for the growth of plants and other organisms. Hence, they are an important part of global biogeochemical cycles [19, 24, 25]. Moreover, the existing coastal microbial composition is often correlated with the carbon–nitrogen recycling and productivity of the ocean, which is a critical criterion for justifying the health of a coastal ecosystem [26–29].

Annual precipitation, climate change, and natural and artificial disasters accelerating loss of native microbial communities and dysfunctional balance in an ecosystem [30]. Determining the connection between the microbiome composition of coastal and plains areas is crucial [14] because it displays clear patterns that highlight the effects of pollution. In our paper, a microbial community analysis from coastal areas and selected forests was carried out. These include long-term observations of changes in coastal areas and certain plains, culture-dependent soil microbiome analysis, high-throughput amplicon sequencing of environmental DNAs, and statistical analyses. India covers a large geographic area in South Asia; therefore, changes in these large geographical areas are substantial over a period of time and are either impacted or governed by recreational activities and domestic and industrial pollution. We considered these factors during the assessment of the soil microbiome using culture-independent, supervised machine-learning and culture-dependent methods to provide valuable insights into the structure and function of microbial communities and identify new taxa over a long period of time. Thus, the study on comparative microbial analysis of coastal regions, Andhra Pradesh vs. Goa, examines the microbial compositions of coastal areas in Andhra Pradesh (AP) and Goa (GA), analyzing the impact of geographic and environmental factors. The hypothesis suggests that factors such as the Bay of Bengal’s characteristics, upwelling, and strong tidal currents influence microbial communities differently in AP and GA. Additionally, the study suggests that variations in temperature, soil type, and vegetation coverage have an impact on these differences in microbial diversity.

## Material and methods

### Sampling site and sample collection

**Fig. 1:**
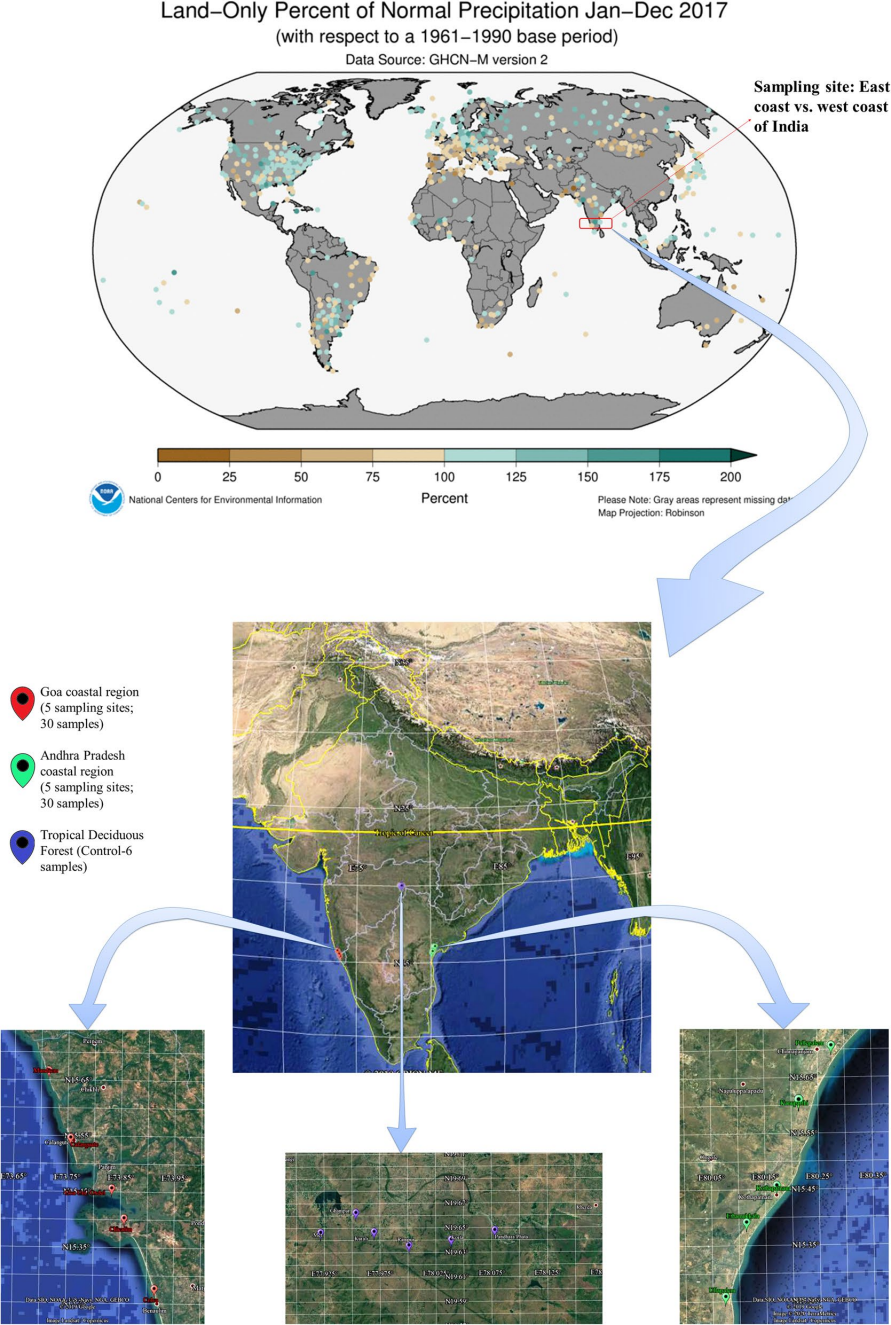
Sampling sites show precipitation in the year 2017. It was recorded that the east coast (approximately 75–100 percent) received more rainfall than the west coast (approximately 100–200 percent) of India. Samples collected from a) Andhra Pradesh’s coastal area were mostly sandy agricultural land, lagoons, and salterns; b) Goa’s coastal area was mostly hilly, and beaches possessed soil, rocks, and sand; and c) the tropical deciduous forest hilly area possessed black soil and Rocky Mountains (Adopted and modified from Source: National Centers for Environmental Information (NCEI) https://www.ncei.noaa.gov/ and Google Earth (https://earth.google.com/))

The coastal areas along the Arabian Sea and the Bay of Bengal were observed for various anthropogenic activities and pollution from 2014 to 2017. Based on firm observations of the coastlines of nine states (West Bengal, Odisha, Andhra Pradesh, Tamil Nadu, Kerala, Karnataka, Goa, Maharashtra, and Gujarat, from east to west), two states, namely, Andhra Pradesh (AP) and Goa (GA) states of India, were selected across the coastal border of mainland India. AP and GA states are situated exactly opposite each other, i.e., sampling sites on coastlines of the Bay of Bengal (from here, Bay) and the Arabian Sea (from here, ASea) are parallel to each other. A total of 80 km of sampling sites were chosen, and coordinates were chosen for composite sampling [31] on the coastlines of AP and GA states (Fig. 1, Video 1). Five sampling sites were chosen for sample collection in each coastal region. The sampling points were chosen in such a way that sampling point 1 on the Bay should be at approximately the same latitude as sampling point 1 on the ASea. The distance between any two sampling sites was approximately 20 km. Metadata from each sampling site were collected. Postmonsoon soil samples were collected in October 2017 from the coastlines of GA, i.e., Colva beach (labeled GCVB01 to 06), Chicalim (GCCL01 to 06), Goa University-Oxdel or Oxdel (GGUO to 06), Calanguate (GCLG to 06), and Mandrem (GMDM01 to 06). Similarly, samples from Pallepalem (APG01 to 06), Ammanbrolu-Kanuparthi (AMB01 to 06), Kothapatanam (AKPT01 to 06), Ethamukkala (AEMK01 to 06), and Ullapalem (AUP01 to 06) in AP were collected. Six control samples from the analysis were collected at border tropical deciduous forests in Nanded District, Maharashtra (from here, Forest), labeled Met (KVRF01), Kurali (KVRF02), Ghampur (KVRF03), Rampur (KVRF04), Korta (KVRF05), and Pandara Phata (KVRF06). A composite sampling method [31] was adopted for sample collection. Ten cm of soil core was collected multiple times by avoiding the humus layer, packed in Nasco Whirl–Pak sampling bags (PW390, HiMedia Laboratories), and transported to the laboratory under refrigerated conditions using dry ice. The maximum temperature recorded during transportation was 5 °C. Samples were subjected to immediate downstream processing, such as cleaning, pH measurement, metagenome extraction and isolation of microorganisms.

### Whole DNA extraction samples and 16S rRNA amplicon sequencing

The whole metagenome was extracted from 66 samples using a Qiagen DNeasy Power Soil Kit (Cat No. 12888-100; 100 preparations) according to the manufacturer’s instructions. A total of 250 mg of soil was used for the extraction of the whole metagenome, i.e., environmental DNA (eDNA), from each sample separately. The eDNA extraction was confirmed using gel electrophoresis, and the DNA quality was measured using a NanoDrop 2000 (Thermo Fisher Scientific, USA), followed by fluorometric quantification using a Qubit (Thermo Fisher Scientific, USA) dsDNA HS assay kit. Then, the extracted DNA was used to create an amplicon library. The V4 region-specific primers (forward 5’ GTGCCAGCMGCCGCGGTAA 3’ and reverse 5’ GGACTACHVGGGTWTCTAAT 3’) [32, 33] and the TaKaRa bacterial 16S rDNA PCR Kit Fast (800) (TaKaRa Bio Inc; Cat. # RR182A) were used to amplify the 16S gene. Prior to polymerase chain reaction (PCR), extracted genomic DNA was diluted to a concentration of 12.5 ng/L. The Applied Biosystems TM 96-Well Thermal Cycler was programmed for PCR using the following parameters: initial denaturation at 95 °C for 3 min; 25 cycles of denaturation at 95 °C for 30 s; annealing at 55 °C for 30 s; and extension at 72 °C for 30 s; and final extension at 72 °C for 5 min. The PCR results were examined for amplification on an agarose gel.

Following amplification, the products were cleaned with AMPure XP beads (A63882, Beckman Coulter, Inc.), and library preparation was performed using the NextraXT DNA Library preparation kit (Illumina, USA) according to the manufacturer’s instructions. To obtain final libraries, final clean-up was conducted using AMPure XP beads, which were then examined for fragment size distribution using TapeStation (5067-5582, Agilent Technologies) and quantified using Qubit DNA (Q32854, Thermo Fisher Scientific) prior to sequencing. The 16S rRNA gene amplicon libraries were sequenced on an in-house Illumina MiSeq platform using paired-end 2 × 250 bp chemistry [34].

### High-throughput sequencing data analysis using in-house bioinformatics pipelines

#### Demultiplexing and denoising in DADA2 and generation of the feature table

Pair-end raw read quality was evaluated with the FastQC [35] tool. Validation of the metadata mapping file was performed using the Keemei tool for standalone Quantitative Insights Into Microbial Ecology 2 (QIIME2) (http://QIIME2.org) of amplicon sequencing data [36–38]. The 16S rRNA gene fastQ forward and reverse reads were processed using QIIME2 version 2023.5 on the macOS M2 Pro M2 platform. QIIME2 was favored for analysis over mothur because it offers more dynamic visualization options [37]. Mothur is more concerned with data generation. Moreover, the inbuilt DADA2 pipeline in QIIME2 was used for quality control processing and to filter any phiX reads and chimeric sequences. The inbuilt dada2 tool in QIIME2 denoised and removed low-quality regions of the sequences. Herein, high-quality bases equal to Q30 (the probability of an incorrect base call is 1 in 1000 and the inferred base call accuracy is 99.9%) were observed around position 250 bases; thus, sequences were truncated at 250 bases. For each amplicon dataset, the error rate was computed. Each dataset was a sequence variant inferred after dereplicating identical readings. Following this, paired-end reads were combined, and chimeras were eliminated. After quality filtering, the resulting data were visualized and summarized for the number of sequences associated with each sample and with each feature. The feature table generated in QIIME2 is called a higher-resolution amplicon variant table, which is analogous to the traditional operational taxonomic unit (OTU) table. All sequences were rarefied to an even sequencing depth of 10,000 sequences per sample to correct for unevenness between samples. Negative control samples were not included in the data analysis because they did not contain suspected contaminants from sampling or PCR amplification.

#### Core-metric, alpha and beta diversity analyses

Alpha and beta diversity analyses were computed by applying related statistical tests, resulting in the generation of interactive visualizations. First, the core-metrics-phylogenetic method was used to rarefy the feature table o that each sample had the same number of features at the same rarefaction depth. This will help to compute several metrics for alpha (Shannon’s diversity index, observed features, Faith’s phylogenetic diversity, and evenness or Pielou’s evenness) and beta (Jaccard distance, Bray‒Curtis distance, unweighted UniFrac distance, and weighted UniFrac distance) diversity and to generate principle coordinate analysis (PCoA) plots using Emperor for each of the beta diversity metrics. Permutational multivariate analysis of variance (PERMANOVA) in QIIME2 calculated differences between microbial communities (beta diversity) based on phylogenetic data displayed on the PCoA plots. To test hypotheses about the differences between groups of data, statistical validation of the analyzed results was performed using ANOVA in QIIME2. In the context of analyzing results with QIIME2, ANOVA can be used to test whether there are significant differences in the microbial community composition between different samples or groups of samples. For example, ANOVA could be used to test whether there are significant differences in the types and abundances of microbes present in samples collected from different locations or under different conditions. Performing statistical validation of the analyzed results using ANOVA in QIIME2 helped to ensure the reliability and robustness of the results. It helps determine whether any observed differences between groups are statistically significant and are not due to random variation or other sources of error. This is an important step in the analysis of microbial community data, as it helps to ensure that the conclusions drawn from the data are reliable and supported by the evidence.

#### Alpha diversity rarefaction

To explore alpha diversity as a function of sampling depth, optionally controlled minimum sampling depth with --p-min-904 and maximum sampling depth with –p-max-95879 were selected, and diversity metrics were computed for all samples in the tables. With --p-iterations, the number of rarefied tables calculated at each sample depth was regulated. Samples can be grouped based on metadata collected at the time of sampling, which results in the visualization of features in each specific group, such as area, place, vegetation, and temperature, with parameters provided in the sample metadata. Average diversity values were plotted for each sample at each sampling depth.

#### Taxonomic analysis

The taxonomic composition of each sample in relation to metadata was calculated by assigning taxonomy to the sequences from feature data using the QIIME2 artifact feature-classifier classify-sklearn. A pretrained Naive Bayes classifier and the q2-feature-classifier plugin (trained on the Greengenes 13_8 99% OTUs) with 250 bases from the V4 region of the 16S rRNA were applied to our sequence data and generated a visualization of the mapped taxonomy. The taxonomic composition of samples with interactive bar plots was visualized on QIIME2 View (https://view.qiime2.org/).

#### Alignment of sequences using MAFFT and phylogeny using FastTree

A phylogenetic tree was constructed from features using the q2-phylogeny artifact (align_to_tree_mafft_fasttree) action without sacrificing scalability in QIIME2. MAFFT was used to remove highly variable positions from a multiple sequence alignment so that an unrooted phylogenetic tree could be made using qiime phylogeny fasttree artifact. Finally, a root will be added to the unrooted tree. The final unrooted phylogenetic tree will be used for analyses that we perform next, specifically for computing phylogenetically aware diversity metrics. The generated tree files were viewed using Interactive Tree of Life v6 [39].

#### Phylogeny with Empress

To confirm the taxonomic categorization made in former steps, analysis with Empress was carried out to investigate the hierarchical relationships between features in a dataset. Empress provides both novel features and functionality [40]. Trees of amplicon sequence variants (ASVs) or operational taxonomic units (OTUs) were created and visualized.

#### Deicode ordination

To test high levels of sparsity, DEICODE (a form of Aitchison Distance) was performed. It offers a robust and relatively simple way to interpret compositional PCA where zero values do not influence the resulting ordination, i.e., compositional biplots. These biplots were visualized in ‘QIIME2 View’ through Emperor [41, 42].

#### Random forest regression and supervised machine learning model

Supervised machine learning approach was used to discriminate the diversity of samples according to the microbiological compositions of the samples. We have provided a benchmark comparison of supervised learning classifiers and regressors that have been built in scikit-learn, which is a machine learning toolkit based on the Python programming language. In addition, we introduce q2-sample-classifier, a plugin for the QIIME2 microbiome bioinformatics platform that makes it easier to apply scikit-learn classifiers to microbiome data. We developed the q2-sample classifier. The models of random forest, additional trees, and gradient boosting perform the best in regard to supervised classification and regression of soil microbiome data [43].

### Isolation and characterization of aerobic heterotrophic bacteria

Selected samples were used for isolation of hydrocarbon and salt-resistant aerobic heterotrophic bacteria [44, 45] on minimal media, R2A media, mannitol salt agar, Zobell Marine Agar, sea water agar, nutrient agar, Luria–Bertani agar (HiMedia Laboratories, Thane), and soil extract agar. Isolation of bacteria was carried out using the spread plate and streak plate methods. The first replica of isolated pure cultures was labeled and preserved in a -80 °C refrigerator at an in-house culture collection facility, i.e., NCMR-NCCS Pune. Colony characteristics, morphological features, resistance to antibiotics, hydrocarbon, and physiological characteristics were recorded.

### Screening and identification of hydrocarbon-resistant, antibiotic-resistant, and slow-growing bacteria using MALD-TOF MS

We decided to profile the most typical bacteria that emerge as resistant in the presence of either hydrocarbons or antibiotics and grow relatively slowly under laboratory conditions on petri plates. Technically, it is very difficult to identify all bacteria using traditional biochemical methods due to their high cost and laborious nature. Bacterial colonies in triplicate were randomly selected from actively growing resistant phenotypes for each sampling site and identified at our in-house MALDI-TOF Biotyper (Bruker Daltonics, Germany) laboratory, as described in the technique [46]. The colonies were spotted on a MALDI target plate, covered with 1:1 matrix solution (alpha-cyano-4-hydroxycinnamic acid - HCCA matrix suspended in 50% acetonitrile and 2.5% trifluoroacetic acid) and allowed to air dry at ambient temperature. The samples were run via the Autoflex speed system (Bruker Daltonik, Germany), and the resulting spectra were utilized to identify bacteria at the species level using MALDI Biotyper software 3.0 (Bruker Daltonik, Germany) against the Bruker proteomics reference library. Only microorganisms with low confidence or unidentified based on their MALDI-TOF score were cross validated using Sanger DNA sequencing [47].

### Confirmation of bacterial identity using 16S rRNA gene sequencing methods

16S rRNA gene sequencing is a method that is commonly used to identify and characterize microbial species. It involves amplifying and sequencing a marker gene, i.e., the 16S ribosomal RNA (rRNA) gene. The 16S rRNA gene is conserved across different species, but there are some differences in the sequence of this gene between species, which can be used to identify and classify them. Using a PCR cycler with the following program: initial denaturation at 94 °C for 4 min, 32 cycles of 94 °C for 25 s, 55 °C for 60 s, and 72 °C for 60 s, followed by a final extension of 4 min at 72 °C, and storage at 4 °C before being sent to an in-house sequencing facility for Sanger sequencing. Each generated sequence was manually analyzed, and contigs were prepared using DNASTAR Lasergene SeqMan bioinformatics tools (https://www.dnastar.com/software/lasergene/). EzBiocloud is a database that contains information on the 16S rRNA gene sequences of a wide range of microbial species. By comparing the sequence of an unknown microbe to the sequences in the EzBiocloud database, it is possible to identify the microbe at the species level or within a related group of species. The resulting contigs were checked for identification of the closest taxa using EzBiocloud (http://www.ezbiocloud.net/).

## Results and discussion

The results showed distinct differences in the microbial community composition of the 66 composite samples in the coastal soil belt, especially between GA and AP, compared with the control (forest).

### Sample sites and highlights of pollution, anthropogenic activities, and recreational activities in coastal areas

Based on the literature and results, it was disclosed that climatic conditions on the two different coastlines are entirely different in terms of water quality and salinity, soil and sand appearance, geographic locations, intrusion of waters, and the natural organization of coastal areas in AP and GA. There are three reasons we discovered over the span of 5 years of observation. First, Bay is a sea located to the east of the Indian subcontinent and north-west of the Indonesian archipelago. It is an important part of the northeastern Indian Ocean and is connected to the ASea to the west (see INCOIS India, incois.gov.in). The Bay is on average 470 feet shallower than ASea [48]. The reduced depth of the Bay may have significant ramifications for the marine ecology. Net primary production (NEP) in the sea, for instance, is equal to the gradual burial of organic matter minus the rate at which organic materials enters continents [49]. This indicates that the majority of organic matter tends to migrate into the deep ocean rather than settling in shallower waters like the Bay, which may have an impact on the microbial composition at the deposit destination and coastal ecosystems.

**Fig. 2:**
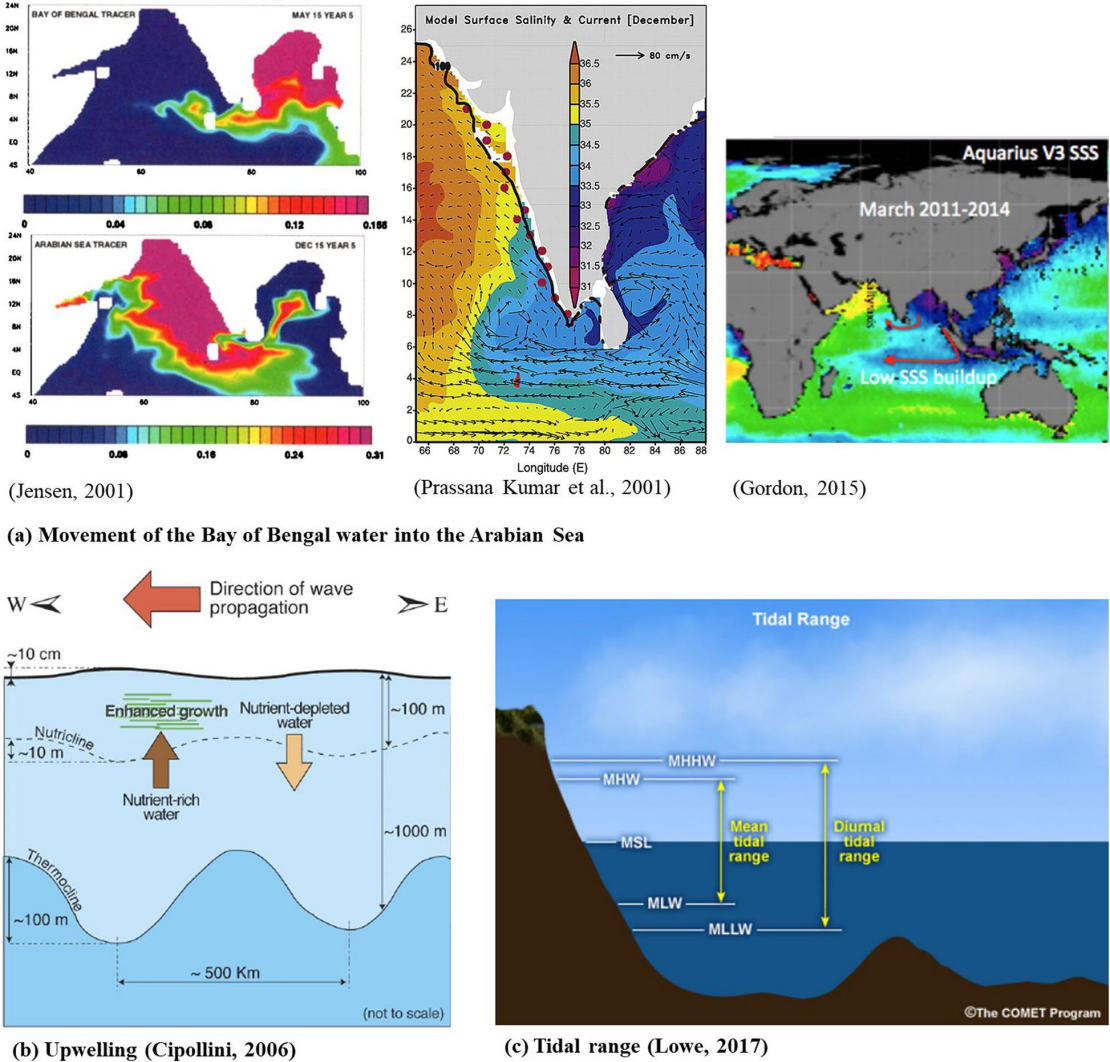
Climatic conditions, geographical structure, and reasons for oil pollution along the Bay and ASea coastlines are: **a** the shallower nature of the Bay and dilution by monsoon water cause a decrease in density and salinity of the Bay water, which results in movement towards the ASea; **b** Upwelling causes upward movement of sunken pollutants; and **c** high tide causes an increase in sea level and drags all pollutants towards beaches across the coastline [50, 51, 53–55]

Moreover, rainwater gathered in the pan of the Bay of Bengal dilutes it and has a natural tendency to flow from the east side to the west side [50, 51] i.e., toward Goa (Fig. 2a). This indicates that salinity of the Bay is reduced due to displacement seawaters. Rengarajan et al. [52] used ^228^Ra and ^226^Ra to study mixing in the surface waters of the western Bay of Bengal. They found that ^228^Ra is a good tracer for figuring out how fast low-salinity waters in the north and high-salinity waters in the south of the western Bay of Bengal mix with each other [52]. This evidence supports our investigation about the movement of oil spills and pollutants due to the intrusion of the Bay water into the ASea because of desalination and the hydrological movement of ocean water.

The second reason for changing coastline characteristics and pollution is the upwelling phenomenon. Upwelling is a phenomenon that occurs when cold, nutrient-rich water from deeper layers of the ocean rises to the surface [56–58]. Upwelling can also occur due to the influence of currents and other oceanic processes [59, 60]. This, in turn, can fuel growth of phytoplankton by delivering chemical nutrients [61] and support the entire marine food web, as these primary producers provide food for a wide variety of animals, including fish, seabirds, and large predators such as whales [62–64]. It can also have global impacts, as upwelling can influence the concentration of carbon dioxide in the atmosphere and bring deeply sunk tar balls and heavy hydrocarbon-rich water to the surface [65, 66]. This allows the exchange of water from the bottom of the ocean to the surface (Fig. 2b).

The third reason is high tide. During high tide, the level of the ocean rises and can reach higher levels than at low tide [67, 68]. This occurs when the gravitational pull of the moon and the sun align in such a way that they increase the gravitational pull on the Earth’s oceans. As the water level rises, it can cover areas of the coastline that are normally above water, including beaches, rocks, and other structures. As the water level rises during high tide, it can also cause waves and currents to strengthen. These waves and currents can drag or push objects that are on the coastline or in shallow water out to sea [69, 70]. The entire coastline of India experiences reverse deposition at beaches as a result of high tide (see Fig. 2c), which drags everything on the sea’s edge and causes movement of everything in and carried by ocean water. We have observed that many live sea animals, such as fish, squids, conches (shankhas), starfish, octopus, unearthed seaweed, algal biomass, and other sunken remnants of ships, boats, and similar objects, were dragged and deposited on the coast after high tide. Photographic evidence (S Fig. 1) shows that the coastline of ASea is more heavily polluted than that of the Bay. Hence, the three reasons mentioned earlier support our investigation and vice versa. Collected evidence proves that the coastal region of Goa was heavily polluted by various pollutants, including (a) oil pollution; (b) a stream of domestic waste dumping into the ASea at Panjim; (c) and (d) plastic pollution at beaches; and (e) used diapers on beaches. These have a huge impact on biodiversity, fauna, and microbial flora residing in marine environments and coastal habitats [71]. Visuals show that many marine animals in huge numbers were found dead on the coastline of the ASea, as mentioned in S Fig. 1: (f) dead crab; (g) alcoholic beverage bottle; (h) dead fish; (i) dead starfish; (j) live octopus; (k) live jellyfish; and (l) dead and rotting squid. We do not find such implications across the AP coastline.

A total of 66 samples were collected from 11 different sites in the AP, GA, and forest areas. Samples were collected from five sites in the AP with an approximate distance of 20 km between the two sample sites. A similar strategy was applied during the collection of samples from the GA coast. Sampling sites in the GA on the west coast were exactly opposite those in the AP along the east coast. Soil samples from the topical deciduous were used as control samples for this study. The composite sampling method was adopted for the collection of coastal samples (see Fig. 1), as mentioned earlier. Six composite samples were collected from each site during the morning hours only. Six composite samples collected from each sampling site were labeled as follows: AEMK01-AEMK06, AKPT01-AKPT06, AMB01-AMB06, APG01-APG06, and AUP01-AUP06 from the AP coastline; GCCL01-GCCL06, GCLG01-GCLG06, GCVB01-GCVB06, GGUO01-GGUO06, and GMDM01-GMDM06 from the GA coastline; and K-series: KVRF01-KVRF06 from forest (see metadata Table 1 and S Fig. 2). Soil collected from the AP and GA coastlines was sandy or loamy in texture. The color of the soil varies but is most commonly black or reddish. However, the soil texture of the control sample was purely organic, loamy and free from pollutants. The environmental temperature during sample collection was typically warm, with an average of 30 °C. Most of the samples were soil, and some were beach sand with different colorations (black, blackish white, red, reddish, whitish black, sandy white, and blackish red). The environmental temperature at the time of sampling ranged from 20 °C to 35 °C (AP), 28 °C to 35 °C (GA), and 30 °C (Forest). Similarly, the pH of the samples was recorded, and most of them were at approximately neutral pH, i.e., 7.0–8.0 (AP), 7.0–7.5 (GA), and 6.5–7 9 (Forest). GA was slightly hotter than AP. Forest cover, agriculture, and minimal intervention in the natural ecosystem could be the reasons for the difference in temperature across these two coastlines. Moreover, the type of soil, temperature, forest cover or vegetation are the most influential factors that lead to changes in the microbial community composition in the coastal soil. Hence, it is necessary to determine community structure characteristics and trace genetic variants found in coastal soil belts [72]. To decipher, the diversity of the microbial community, 16S rRNA amplicon sequencing of the above collected samples was carried out, followed by bioinformatics analysis using QIIME2.

**Table 1:** Metadata of samples collected for 16S rRNA amplicon sequencing and bioinformatics analyses from coastlines of AP and GA, and Forest

**Area**	**Place**	**Sample type**	**Color**	**Sample ID**	**Coordinates**		**Environ. Temp. (**^**o**^**C)**	**pH**
					**Latitude**	**Longitude**		
Andhra Pradesh coastline	Ethamukkala	Soil	Blackish white	AEMK01	15°22′52.6″	80°06′10.8″	30	7
			Black	AEMK02	15°22′52.5″	80°06′11.1″	32	8
				AEMK03	15°22′52.1″	80°06′11.4″		
				AEMK04	15°22′51.9″	80°06′10.3″	32	7
				AEMK05	15°22′51.5″	80°06′10.0″	30	7
				AEMK06	15°22′50.4″	80°06′09.7″	32	7.5
	Kothapatnam	Soil	Black	AKPT01	15°26′26.3″	80°08′45.3″	25	8
				AKPT02	15°26′26.0″	80°08′45.8″		
				AKPT03	15°26′25.1″	80°08′45.8″		7.5
				AKPT04	15°26′09.9″	80°10′40.0″		
			Blackish white	AKPT05	15°26′09.2″	80°10′38.3″		
			Red	AKPT06	15°26′09.2″	80°10′38.4″		7
	Ammanbrolu and Kanuparthi	Soil	Reddish	AMB01	15°35′39.8″	80°08′43.4″	20	7.5
				AMB02	15°35′40.0″	80°08′42.9″		
				AMB03	15°35′40.5″	80°08′43.9″		
			Whitish	AMB04	15°34′44.4″	80°13′06.2″	23	
				AMB05	15°34′23.4″	80°13′20.4″		
			Whitish black	AMB06	15°34′23.5″	80°13′19.9″		8
	Pallepalem	Sandy soil	Black	APG01	15°41′22.4″	80°16′56.0″	20	8
			Sandy white	APG02	15°41′22.7″	80°16′57.0″		7.5
			Black	APG03	15°41′22.0″	80°16′56.7″		
		Beach sand	Sandy white	APG04	15°41′17.3″	80°17′04.8″		8
		Soil	Whitish black	APG05	15°41′32.5″	80°15′54.03″		
			Whitish black	APG06	15°41′32.2″	80°15′54.3″		7.5
	Ullapalem	Soil	Light black	AUP01	15°14′15.0″	80°03′05.6″	35	7
				AUP02	15°14′15.1″	80°03′04.3″		
			Reddish	AUP03	15°14′14.7″	80°03′05.0″		
			Yellowish	AUP04	15°14′13.5″	80°03′05.6″		7.5
				AUP05	15°14′13.4″	80°03′05.6″		
			Black	AUP06	15°14′12.3″	80°03′05.8″		8
Goa coastline	Chicalim	Soil	Black	GCCL01	15°23′56.4″	73°50′50.0″	25	7
				GCCL02	15°23′55.9″	73°50′49.9″		
				GCCL03	15°23′55.5″	73°50′49.9″		
			Blackish	GCCL04	15°23′52.2″	73°50′51.6″		7.5
			Black	GCCL05	15°23′90.2″	73°54′78.5″		
				GCCL06	15°23′90.2″	73°54′81.4″		
	Calanguate	Soil	Reddish black	GCLG01	15°31′54.1″	73°45′48.7″	32	7
				GCLG02	15°31′54.5″	73°45′48.3″		
				GCLG03	15°31′54.7″	73°45′49.0″		
				GCLG04	15°31′53.1″	73°45′49.2″		
			Red	GCLG05	15°31′53.9″	73°45′49.0″		
				GCLG06	15°31′54.0″	73°45′48.4″		
	Colva	Beach sand	Sandy white	GCVB01	15°16′40.5″	73°54′44.5″	29	7
				GCVB02	15°16′42.0″	73°54′47.3″		7.5
				GCVB03	15°16′42.5″	73°54′47.7″		7
				GCVB04	15°16′42.4″	73°54′48.0″		7.5
				GCVB05	15°16′40.5″	73°54′48.2″		7
				GCVB06	15°16′41.4″	73°54′47.2″		
	Oxdel	Soil	Blackish red	GGUO01	15°27′18.2″	73°50′04.2″	25	7
			Red	GGUO02	15°27′18.9″	73°50′09.8″		
			Blackish	GGUO03	15°27′18.8″	73°50′15.1″		
				GGUO04	15°27′14.2″	73°50′19.0″		
				GGUO05	15°27′10.7″	73°50′19.0″		
				GGUO06	15°27′08.9″	73°50′20.9″		
	Mandrem	Soil	Reddish	GMDM01	15°39′59.1″	73°42′54.0″	28	7
			Black	GMDM02	15°39′59.2″	73°42′54.9″		
			Reddish	GMDM03	15°40′20.5″	73°42′35.6″		
			Blackish red	GMDM04	15°39′37.1″	73°44′38.2″		
				GMDM05	15°39′36.2″	73°44′38.4″		
				GMDM06	15°39′36.5″	73°44′37.5″		
Forest	Met	Soil	Black	KVRF01	19°38′22.8″	77°55′18.1″	30	6.5
	Kurali			KVRF02	19°38′24.5″	77°58′10.7″		7
	Ghamapur			KVRF03	19°38′22.3″	78°58′10.6″		
	Rampur			KVRF04	19°37′44.2″	78°00′02.9″		
	Korta		Yellowish black	KVRF05	19°37′47.0″	78°03′72.7″		7.5
	Pandhra Phata		Black	KVRF06	19°38′27.9″	78°04′39.8″		7

#### Amplicon sequencing of the V4 region and bioinformatics analysis

**Fig. 3:**
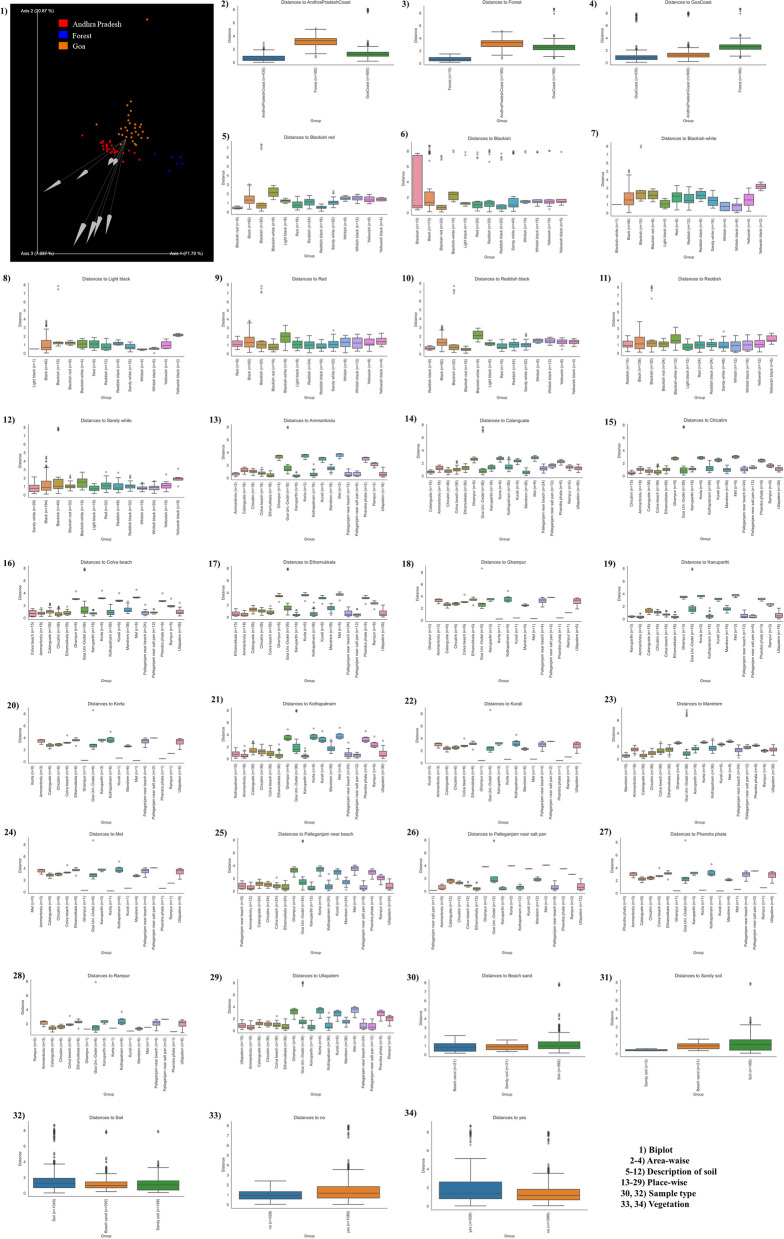
Deicode ordination - Measurement of Aitchison distance to find levels of sparsity in deicode ordination to understand beta diversity analysis

**Fig. 4:**
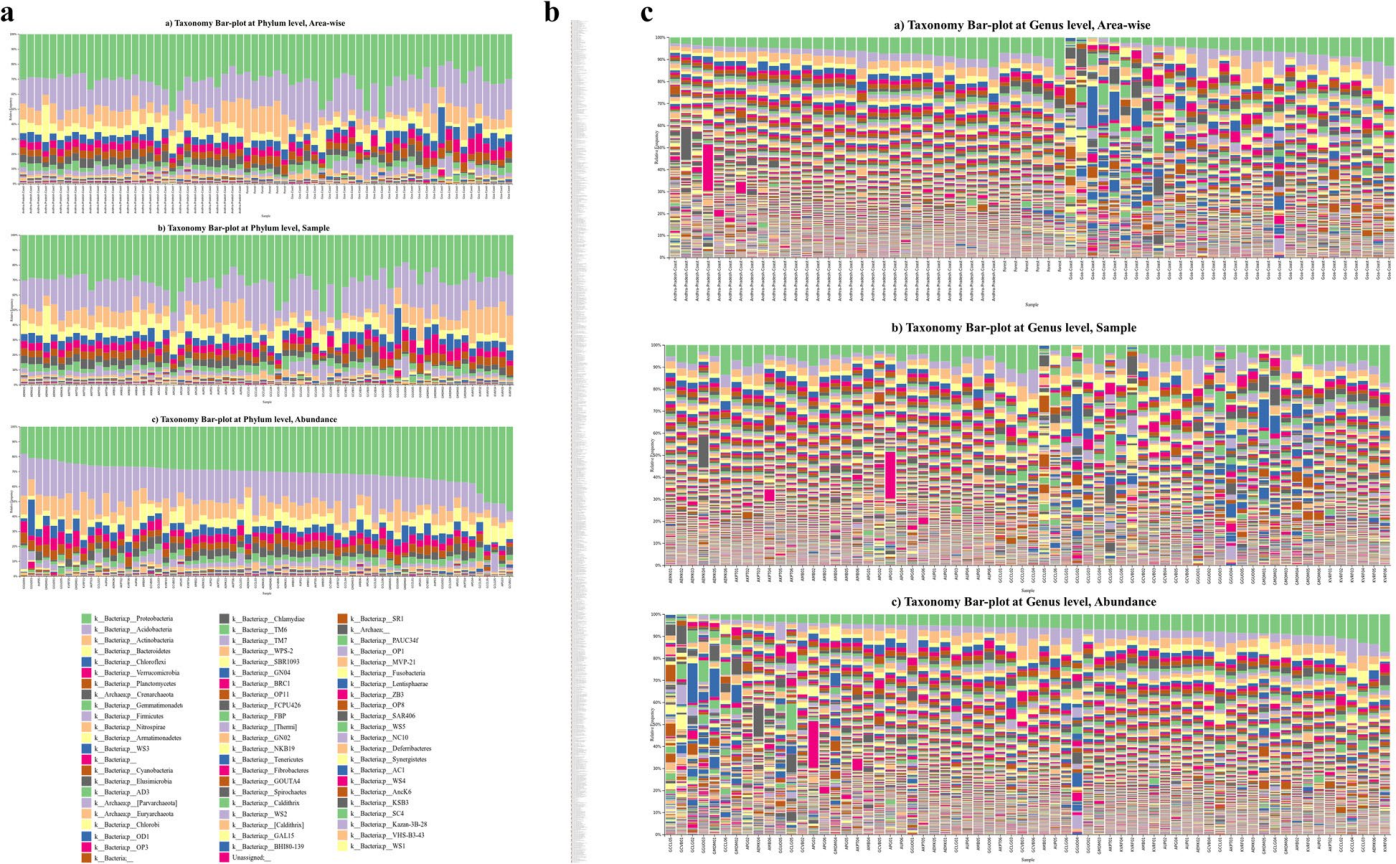
Taxonomic analyses- Bar plots represents phylum level (**a**) and genus level (**b**) distribution of microbial taxa in soil of Goa, A. P. and Forest

The 16S amplicon sequencing and bioinformatic analysis using QIIME2 revealed features found in 66 composite samples of environmental DNA. Demultiplexing of sequences yielded 91, 25, and 440 operational taxonomic units (OTUs) for taxonomic classification with 559607, 145479.5, 138264, and 3024 OTU maximum, median, mean, and minimum values, respectively (S Tables 1 and 2). Additionally, we removed truncated reads, reads that were too short after transcription, and reads that had more than the maximum number of ambiguous bases during denoising of samples using DADA2 true 91,24,744 quality reads for taxonomic classification, with an average of 1,38,253.6 per sample (S Table 3). A feature classifier sklearn A total of 61,22,782 frequencies contained 50,480 operational taxonomic units (OTUs) (S Table 4). Sequence length statistics and a seven-number summary of sequence lengths indicate that among more than 50,000 OTUs, the mean length is 227.19 ± 3.3 bases, with 98% of sequences being 229 bases long. Of the total sequences sampled at a depth of 10,000, 98 percent of forward sequence OTUs and 89 percent of reverse sequence OTUs were 251 nt long, with 50 percent having a median value of 251 nt and median frequency of 95,879 (S Tables 5 and 6). Box and whisker plots of the number of OTUs per sample indicate the possibilities of diverse microbial communities in each sampling zone. Interactive OTU plots were generated using a random sampling of 10,000 out of 9,125,440 sequences without replacement. This position (251) is greater than the minimum sequence length observed during subsampling (248 bases). As a result, the plot at this position is not based on data from all of the sequences, so it should be interpreted with caution when compared to plots for other positions (S Fig. 3). Multiple taxonomic levels, such as phylum and species, were used to classify representative sequences from our dataset. Sequences were normalized for use in subsequent QIIME2 analyses. A real-time analysis of the soil microbiome revealed that a total of 59,664 (0.97%) features in 66 (100.00%) samples and 650,000 (10.62%) features in 65 (98.48%) samples were retained at sampling depths of 904 and 10,000, respectively (S Fig. 4, S Tables 7.1 and 7.2). Understanding coastal ecosystems remains a challenging task due to frequent changes in climatic conditions, the exchange of soil‒water microbiomes, and the impact of pollutants, especially oil spills and hazardous recalcitrant compounds.

The q2-diversity plugin inferred plugin core-metrics-phylogenetic rarefied feature table and MAFFT tools insightful phylogenetic FastTree (Fig. S5a and b). It was seen that most of the circular rooted and unrooted trees have a high number of frequencies that cause related child nodes to group together on sister nodes. Taxonomy was automatically assigned using the Genome Taxonomy Database (GTDB). Moreover, soil from GA, AP, and forest areas harbors bacterial phyla from Candidatus Phyla Radiation (CPR). In alpha diversity analysis, Pielou’s evenness showed that microbial communities inhabiting samples from GA (average Pielou evenness = 0.94) were more diverse than those from the AP coast (average Pielou evenness = 0.96). However, the diversity in the forest samples was relatively conserved (average Pielou evenness = 0.97). The Kruskal‒Wallis test (nonparametric ANOVA) found a significant high difference among all groups. *P* value indicates (*p* = 0.001) ndicating that the microbial communities inhabiting soil collected from GA, AP, and Forest were significantly different (S Fig. 6, S Tables 8 and 9). Furthermore, alpha rarefaction evidence from Faith PD, observed OTUs, and Shannon index values indicates subset selection, and the calculation of alpha diversity for a randomly selected subset at a depth of 904 sequences in each sample confirms that sequencing was deep enough and captured most of the diversity from the collected samples. Alpha diversity increased significantly in GA compared to AP and Forest as measured by the Simpson index (P 0.001) (S Fig. 7, and S Tables 10.1-10.3). Bray‒Curtis, Jaccard distance, unweighted and weighted UniFrac, and permutational multivariate analysis of variance (PERMANOVA) tests were used to analyze the overall bacterial community composition between samples (AP, GA, and forest) and found significant differences between communities (*p* value = 0.001 (area) and *p* value = 0.002 (description of soil)). Principal component analysis (PCoA) reveals a consistent structure and distribution pattern across a wide variety of biologically diverse communities. This pattern can be seen across all of the communities in different PCoAs (Fig. S8a–d and S Table 11). Samples taken from two distinct environments, namely, the AP and the forest, came together to form a distinct cluster. On the other hand, samples taken from the GA area have been divided into four groups, one of which has some similarities with the samples taken from the AP and the forest. Unweighted UniFrac, which rates differences from 0 to 1 (0 being no difference and 1 being a complete difference), indicates a significant difference. In contrast to the difference between the AP and forest communities, which is almost the same at 0.65 0.05 (S Fig. 8e), the diversity difference between the GA communities ranges from 0.85 to 9.0. Additionally, another beta diversity plot with a description of the soil supports earlier findings that soil from the GA area supports more diverse microbial communities than AP and Forest, including blackish, blackish-red, sandy-white, reddish-black, and reddish. Microbial communities from the AP and forest, however, were more conserved or resembled one another (S Fig. 8f). Additionally, Aitchison distance was used to find high levels of sparsity in the deicode ordination and supported the results of beta diversity analysis (Fig. 3). The fact that the compositional biplot log ratio of the GA samples points in a different direction than those of the AP and Forest samples shows that the beta diversity features were correct.

The taxonomic analysis of communities inhabiting coastal soil and forest environments comprised a total of 68 phyla. Proteobacteria (32%), followed by Acidobacteria (18%), Actinobacteria (11%), Planctomycetes (6%), Bacteroidetes (7%), Verrucomicrobia (6%), Chloroflexi (6%), Thaumarchaeota (3%), Firmicutes (2%), Gemmatimonadetes (2%), Robubacteria (1%), Latescibacteria (1%), Elusimicrobia (1%), and Nitrospirae (1%), which contributed up to 97% of the total community of the soil samples. Within the phylum Proteobacteria, Gammaproteobacteria (41.092%) was the most abundant class, followed by Alphaproteobacteria (37.594%), Deltaproteobacteria (21.21%), Betaproteobacteria (0.103%) and Zetaproteobacteria (0.00193%). were detected at relatively lower abundances (< 1%) in the samples (Fig. 4a). Microorganisms are the most ancient constituents of the ecosystem in the Earth’s surroundings [73] and are subjected to frequent changes or modifications. Changes in the chemical compositions of the natural environment are always triggered by human-made activities, which may not have an adverse impact on higher organisms but definitely change the native microbiome composition [74, 75]. This indicates that the bacterial community in soil is highly diverse and that there are significant differences in the community composition between different geographical regions. These findings have important implications for our understanding of the role of bacteria in soil ecosystems and for the development of sustainable agricultural practices [76]. Furthermore, the relative abundance of taxa at the genus level also indicates huge diversity in the case of samples from the GA coast, such as GCCL05, GCVB01, GCLG02, GGUO03, GMDM01, GCCL06, GMDM02, GGUO05, GCLG05, and GCVB02; from the AP coast, such as APG02, AEMK04, AMB06, and APG03; and from the forest, such as KVRF01 and KVRF06. The acidobacterial group Ellin6075 was significantly more prevalent in APG03 soil samples (Fig. 4b). Several members of Candidate Phyla Radiation (CPR) were detected, including Robubacteria (1%) and Latescibacteria (1%), contributing to a large diversity of bacteria [76]. The EMPress tree of amplicon sequence variants (ASVs), which represents evolutionary relationships between OTUs, was used to derive the taxonomic hierarchy between OTUs. To represent evolutionary dominance, Asgardaeota, Elusimicrobia, Proteobacteria, Thermoachaeota, Acetothermia, Dependentiae, and Firmicutes were representative phyla. Other bacterial phyla that made significant contribution in a huge diversity belongs to *Acetothermia, AncK6, Armatimonadetes, Asgardaeota, Calditrichaeota, Chlamydiae, Crenarchaeota, Cyanobacteria, Dadabacteria, Deferribacteres, Deinococcus-Thermus, Dependentiae,Diapherotrites, Elusimicrobia, Entotheonellaeota, Epsilonbacteraeota, Euryarchaeota, Fibrobacteres, Fusobacteria, Gemmatimonadetes, Halanaerobiaeota, Hydrogenedentes, Hydrothermae, Hydrothermarchaeota, Incertae Sedis, Kiritimatiellaeota, Lentisphaerae, Margulisbacteria, Modulibacteria, Nanoarchaeaeota, Nitrospinae, Omnitrophicaeota, Opisthokonta, Patescibacteria, Schekmanbacteria, Spirochaetes, Synergistetes, Tenericutes*Phyla such as GAL15, BRC1, CK-2C2, FBP, FCPU426, PAUC34f, WOR-1, WOR-2, WS1, WS2, WS4 and some unspecified represented a huge number of candidatus phyla (Fig. 4a and S Fig. 9).

### Random forest regression and supervised machine learning model for spotting trend in development of microbiome coastal areas

**Fig. 5:**
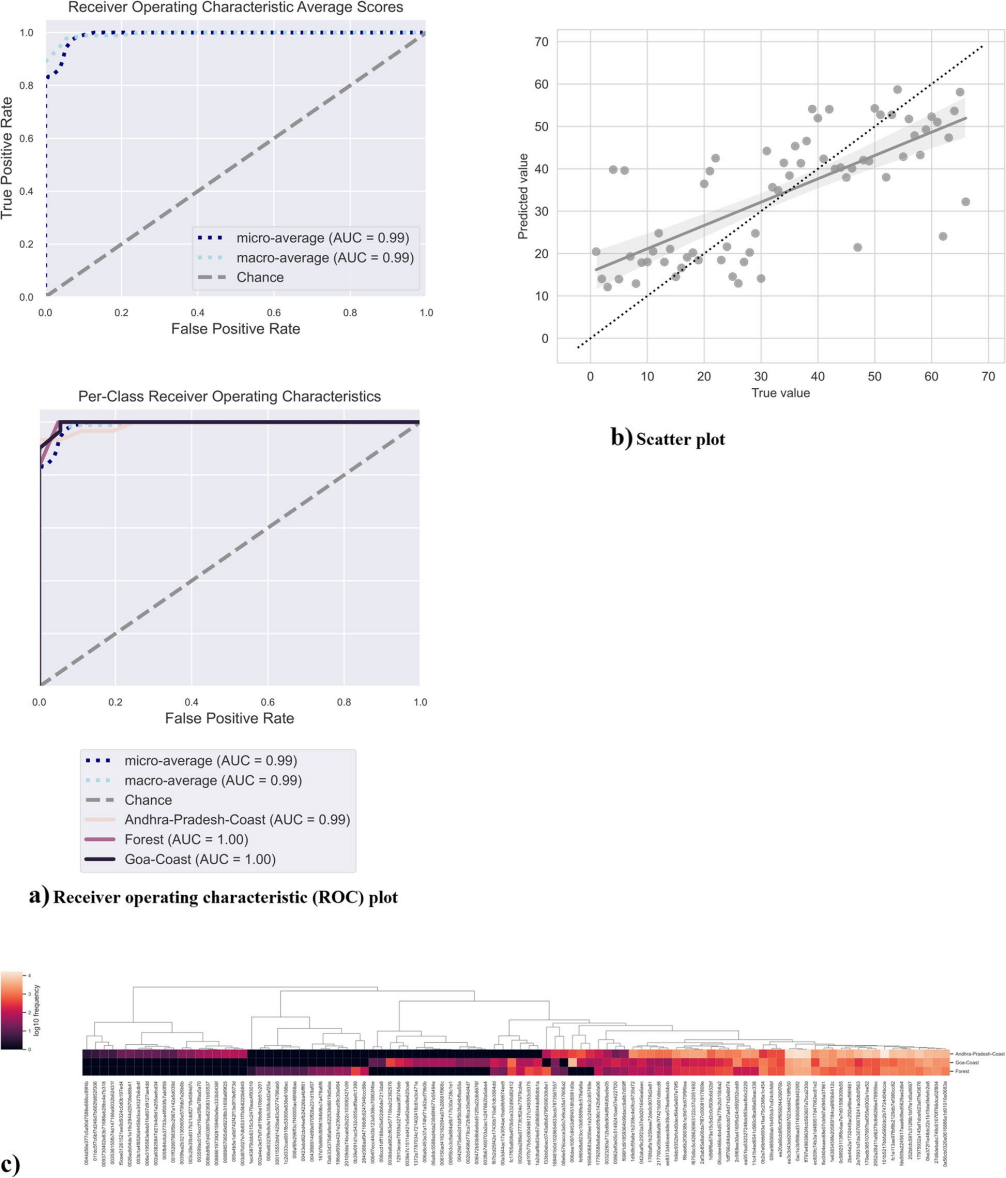
Receiver Operating Characteristic (ROC) curves - **a** The ROC curve plots the relationship between the true positive rate (TPR, on the y-axis) and the false positive rate (FPR, on the x-axis) at various threshold settings. The line on the top-left corner of the plot indicates GA soil diversity, the pink line indicates forest diversity, and the light orange line indicates AP soil diversity. Thus, the top-left corner of the plot represents the “optimal” performance position, indicating a FPR of zero and a TPR of one. This “optimal” scenario is unlikely to occur in practice, but a greater area under the curve (AUC) indicates better performance. This can be compared to the error rate achieved by random chance, which is represented here as a diagonal line extending from the lower-left to upper-right corners. Additionally, the “steepness” of the curve is important, as a good classifier should maximize the TPR while minimizing the FPR. In addition to showing the ROC curves for each class, average ROCs and AUCs are calculated. “Micro-averaging” calculates metrics globally by averaging across each sample; hence class imbalance impacts this metric. “Macro-averaging” is another average metric, which gives equal weight to the classification of each sample. **b** Scatter plot: Linear regression scatter plots (for regression) of predicted and expected classes/values for soil microbiome. **c** Supervised machine learning generated a heatmap of the top 100 taxa. All taxa that contributed to the majority of the sequences among the sample belonged to Candadatus Phyla Radiation

A supervised machine learning method was used to examine and spot trends in microbiome data using the QIIME2 artifact q2-sample classifier. To select the features that provide the highest possible level of prediction accuracy, a process known as feature selection is carried out. This process makes use of cross-validated recursive feature elimination. In addition, regression analysis and classification model studies were carried out. The AP dataset is the simplest; it can be differentiated clearly on PCoA plots and shows significant intrasample similarity between samples from the same area, whereas other datasets display a higher spread and can be differentiated from GA and forest samples. The AP dataset is the simplest. It can be differentiated visually on PCoA plots, as mentioned earlier, and the new confusion matrix plot (S Fig. 10) created using random-forest regression. On a scale of 0 to 1, 0 indicates diversity, whereas 1 indicates uniqueness. Based on these parameters, the overall accuracy of the microbiome was 0.9, whereas the baseline accuracy was 0.45. The repressor accuracy results for AP coastal soil were 0.9, followed by forest at 0.66. On the other hand, GA coastal soil has shared diversity closer to forest, i.e., 0.33, than AP soil, i.e., 0.033, and vice versa. This indicates that GA soil harbors a diverse microbial community and is mostly different from AP soil, whereas forest soil diversity is more inclined toward AP soil [77, 78]. Moreover, a machine learning model was applied to study classification accuracy. Receiver operating characteristic (ROC) curves are a graphical representation of the classification accuracy of a machine-learning model. A machine-learning model’s classification accuracy is graphically depicted using receiver operating characteristic (ROC) curves. The ROC curve illustrates, for various threshold values, 0.99 for AP and 1 for GA and Forest. Hence, GA coastal soil has a greater area under the curve (AUC), indicating better diversity than AP coastal soil (Fig. 5 and b). Forest diversity is steep toward the AP coastal soil, confirming the accuracy of the obtained results. Hence, the results of the supervised machine learning and random forest regression models were visualized through a heatmap of randomly selected taxa, almost all of which represented Candidatus (Fig. 5c). The log_10_ frequency (0–4) of features was normalized and represents dark blue (0) to light orange (4). The degree and direction of the connection is represented by the color of each cell; cells with darker colors have greater correlations, whereas lighter hues have weaker correlations [79].

### Isolation and identification of heterotrophic bacteria

Culture-based investigations have been carried out for further clarification using harsh chemicals and stringent protocols. More than 450 bacteria were isolated and screened for their identification using MALDI-TO MS in-house at NCMR-NCCS Pune. Based on MALDI-TOF MS results and resistance profiles, 134 bacteria were selected for further confirmation of identification. 16S rRNA gene sequence analysis and identification using EzBiocloud suggests that isolated strains are extremely diverse and possess biotechnological applications. Inference was derived from characteristics and published reports. These taxa were distributed among 21 different genera, including *Achromobacter denitrificans*,* Aspergillus quadralineatus*, *Azohydromonas australica*, *Azotobacter chroococcum*, *Bacillus filamentous*, *Bacillus marisflavi*, *Bacillus mycoides*, *Bacillus pacificus*, *Bacillus paralicheniformis*, *Brevibacterium casei*, *Brvundimonas albigilva*, *Cellulosimicrobium cellulans*, *Domibacillus indicus*, *Lysobacter soli*, *Microbacterium telephonicum*, *Mycobacterium spp. LZMCs*, *Mycolibacterium pallens*, *Nomomuraea candida*, *Rhodococcus equi*, *Salinicola salarius*, *Sphingomonas dessicabilis*, *Staphyllococcus gallinarium*, *Thalassobacillus hwangdonitrificans* (one strain each), *Virgibacillus halodenitrficans*, *Acinetobacter towneri*, *Bacillus licheniformis*, *Chitinophaga rhizospaerae*, *Oceanobacillus kimchii* (two strains each), *Bacillus subtilis subsp. stericoris*, *Lysobacter panacisoli* (three strains each), *Bacillus firmus* (four strains), *Bacillus aryabhattai* (seven strains) and *Bacillus paramycoides* (eight strains). The existence of huge diversity represented by 21 genera in culture-based identification shows that collected samples were rich in microbial flora, with huge biotechnological potential [80, 81]. Metagenome analysis reveals the presence of bacteria, like *Mycobacterium* spp., *Vibrio* spp., *Pseudomonas* spp., *Aeromonas* spp., and others, are more common and cause bacterial diseases in fish [82]. Later, these may be lethal causes for sea food-borne infections in humans through ingestion [83, 84].

### Novel taxa and deposition of 16D rRNA gene sequences of heterotrophic bacteria

A few strains were identified as novel strains based on culture-dependent analysis and sequencing: *Chitinophaga caseinilyticastrain* strain GCCLKN05(OQ975925), *Priestia filamentosa* strain AMBL002 (OQ975923), *Chitinophaga caseinilytica* strain MSPCSM02 (OQ975935), *Bacillus paranthracis* strain TYGMDM05 (OQ975939), *Acinetobacter towneri* strain TYGCLG05 (OQ975938), *Domibacillus indicus* strain SEGGUO07 (OQ975936), *Rossellomorea marisflavi* strain GCLG005 (OQ975926), *Acinetobacter towneri* strain TYGCCL04 (OQ975937), *Thalassobacillus hwangdonensis* strain AMBL003 (OQ975924), *Bacillus zanthoxyli* strain GCVB002 (OQ975927), *Bacillus mobilis* strain GOACSMMS16 (OQ975934), *Bacillus paramycoides* strain GOAA7MS06 (OQ975928), *Bacillus velezensis* strain GOAAR2A13 (OQ975931), *Bacillus infantis* strain GOABTMNBNR19 (OQ975932), *Bacillus paramycoides* strain GOAAMS05 (OQ975929), *Bacillus paramycoides* strain GOAAR2A07 (OQ975930), and *Bacillus cereus* strain GOACSMMS11 (OQ975933) were identified and shows 96.22, 96.47, 97.53, 97.94, 98.32, 98.34, 98.35, 98.57, 99.07, 99.16, 99.37, 99.38, 99.44, 99.65, 99.93, 100, 100% similarity, respectively, with standard type strains (Table 2). Thus, identification of the 16S rRNA gene using the EzBioclud database indicates that GCCLKN05, AMBL002, MSPCSM02, TYGMDM05, TYGCLG05, SEGGUO07, GCLG005, and TYGCCL04 are novel taxa at least at the genus or species level. The DNA sequences of these bacteria have been deposited in the NCBI Genebank with accession numbers ranging from OQ975923 to OQ975939. Identification of novel strains is necessary for a number of reasons. First, it can help us to better understand the cultivable diversity of the soil microbiome [85, 86]. Second, it can lead to the discovery of new enzymes and other biomolecules with potential applications in biotechnology [87, 88]. Third, it can help us to understand the role of bacteria and archaea in soil health and nutrient cycling [89–91]. The identification of novel strains is often a challenging task [92, 93]. This is because the soil microbiome is extremely diverse, and many strains are difficult to culture in the laboratory. However, advances in sequencing technology have made it easier to identify novel strains. The identification of a few novel strains based on culture-dependent analysis and sequencing is a significant finding. This suggests that the soil microbiome is even more diverse than previously thought and that there are still many novel strains to be discovered [94–97]. This finding has important implications for our understanding of soil health and nutrient cycling, and it could lead to the discovery of new biomolecules with potential applications in biotechnology [98].

**Table 2:** 16S rRNA gene sequence identification of isolated heterotrophic pure cultures

**NCBI Accession No.**	**Sequence ID**	**Top-hit taxon**	**Top-hit strain**	**Similarity (%)**	**Completeness (%)**
OQ975923	AMBL002	*Priestia filamentosa*	SGD-14	96.47	96.7
OQ975924	AMBL003	*Thalassobacillus hwangdonensis*	AD-1	99.07	94
OQ975925	GCCLKN05	*Chitinophaga caseinilytica*	S-52	96.22	92.3
OQ975926	GCLG005	*Rossellomorea marisflavi*	JCM 11544	98.35	95.3
OQ975927	GCVB002	*Bacillus zanthoxyli*	1433	99.16	97.5
OQ975928	GOAA7MS06	*Bacillus paramycoides*	NH24A2	99.38	87.9
OQ975929	GOAAMS05	*Bacillus paramycoides*	NH24A2	99.93	94.7
OQ975930	GOAAR2A07	*Bacillus paramycoides*	NH24A2	100	96.3
OQ975931	GOAAR2A13	*Bacillus velezensis*	CR-502	99.44	87.5
OQ975932	GOABTMNBNR19	*Bacillus infantis*	NRRL B-14911	99.65	97.9
OQ975933	GOACSMMS11	*Bacillus cereus*	Rock3-44	100	98.4
OQ975934	GOACSMMS16	*Bacillus mobilis*	0711P9-1	99.37	97.4
OQ975935	MSPCSM02	*Chitinophaga caseinilytica*	S-52	97.53	96.8
OQ975936	SEGGUO07	*Domibacillus indicus*	SD111	98.34	96.9
OQ975937	TYGCCL04	*Acinetobacter towneri*	DSM 14962	98.57	96.2
OQ975938	TYGCLG05	*Acinetobacter towneri*	DSM 14962	98.32	96.2
OQ975939	TYGMDM05	*Bacillus paranthracis*	Mn5	97.94	96.5

## Conclusion

This study investigated the coastal areas of Andhra Pradesh (AP) and Goa (GA) and compared them for the presence of pollution and microbiome. Three primary reasons for these differences were identified: the shallower depth of the Bay of Bengal leading to different water movement patterns, upwelling phenomena influencing nutrient-rich water at the surface, and the impact of high tides on coastal dynamics. This study provided insights into the pollution levels and microbial diversity of coastal soil. The analysis of amplicon sequencing data and bioinformatics tools revealed a diverse microbial community in both regions. GA coastal soil showed higher diversity than AP, while forest soil had similarities to AP soil. The supervised machine-learning model further confirmed the distinction between the three regions based on their microbial diversity. Culture-based investigations resulted in the isolation and identification of numerous heterotrophic bacteria from the coastal soil, with several strains identified as novel taxa. These findings indicate the richness of microbial flora potential biotechnological applications in the sampled coastal areas. Hence, this study sheds light on the environmental status and microbial diversity of coastal regions in AP and GA, providing valuable information for further research and environmental management efforts.

### Supplementary Information

**Additional file 1: Video 1.** Sampling site descriptions show geographical locations, arrangements, and vegetation cover.

**Additional file 2.**

**Additional file 3.**

**Additional file 4.**

**Additional file 5.**

**Additional file 6.**

**Additional file 7.**

**Additional file 8.**

**Additional file 9.**

**Additional file 10.**

**Additional file 11.**

**Additional file 12.**

**Additional file 13.**

## Data Availability

The datasets generated and/or analysed during the current study are available in the National Centre for Biotechnology Information (NCBI), The 16S rRNA amplicons in the Sequence Read Archive (SRA) under BioProject PRJNA987149 can be found at the NCBI using the accession numbers SRR25014910 to SRR25014975 with BioSample (SAMN35877910 to SAMN35877939, SAMN35877974 to SAMN35878003, and SAMN35878078 to SAMN35878083). Metadata and OTU table data are available for future analysis at https://github.com/MicrobeAI/goaapfobiome.git.
