# Correction: A culture‑independent approach, supervised machine learning, and the characterization of the microbial community composition of coastal areas across the Bay of Bengal and the Arabian Sea

**DOI:** 10.1186/s12866-024-03382-6

**Published:** 2024-06-22

**Authors:** Bhagwan Narayan Rekadwad, Yogesh Shreepad Shouche, Kamlesh Jangid

**Affiliations:** 1 National Centre for Microbial Resource, DBT - National Centre for Cell Science (DBT-NCCS), Savitribai Phule Pune University (SPPU), Campus, Ganeshkhind Road, NCCS‑Complex, Pune, 411007 Maharashtra India; 2 MicrobeAI Lab, Division of Microbiology and Biotechnology, Deemed to be University), Yenepoya Research Centre, University Road, Yenepoya, Deralakatte, Mangalore, 575018 Karnataka India; 3 Bioenergy Group, DST-Agharkar Research Institute, Gopal Ganesh Agarkar Road, Pune, 411 004 Maharashtra India; 4 Gut Microbiology Research Division, SKAN Research Trust, Bangalore, 560034 Karnataka India

**Correction**: ***BMC Microbiol*24, 162 (2024)**

10.1186/s12866-024-03295-4

Following publication of the original article [1], the authors would like to change the primary affiliation of Dr. Yogesh S. Shouche to “National Centre for Microbial Resource, DBT - National Centre for Cell Science (DBT-NCCS), NCCS Complex, Savitribai Phule Pune University (SPPU) Campus, Ganeshkhind Road, Pune, Maharashtra 411007, India,” instead of “MicrobeAI Lab, Division of Microbiology and Biotechnology, Yenepoya Research Centre, Yenepoya (Deemed to be University), University Road, Deralakatte, Mangalore, Karnataka 575018, India”.

## References

[CR1] 1. Rekadwad, B.N., Shouche, Y.S. & Jangid, K. A culture-independent approach, supervised machine learning, and the characterization of the microbial community composition of coastal areas across the Bay of Bengal and the Arabian Sea. *BMC Microbiol* **24**, 162 (2024). https://doi.org/10.1186/s12866-024-03295-4 10.1186/s12866-024-03295-4 PMC11084130 38730339

