# Supplementary material for: A culture-independent approach, supervised machine learning, and the characterization of the microbial community composition of coastal areas across the Bay of Bengal and the Arabian Sea
Source: BMC Microbiol. 2024 May 10;24:162. doi: 10.1186/s12866-024-03295-4 (PMC11084130; doi:10.1186/s12866-024-03295-4)
Supplement: Supplementary file 2 — Additional file 2. [file 12866_2024_3295_MOESM2_ESM.doc]

**Legend to supplementary figures**


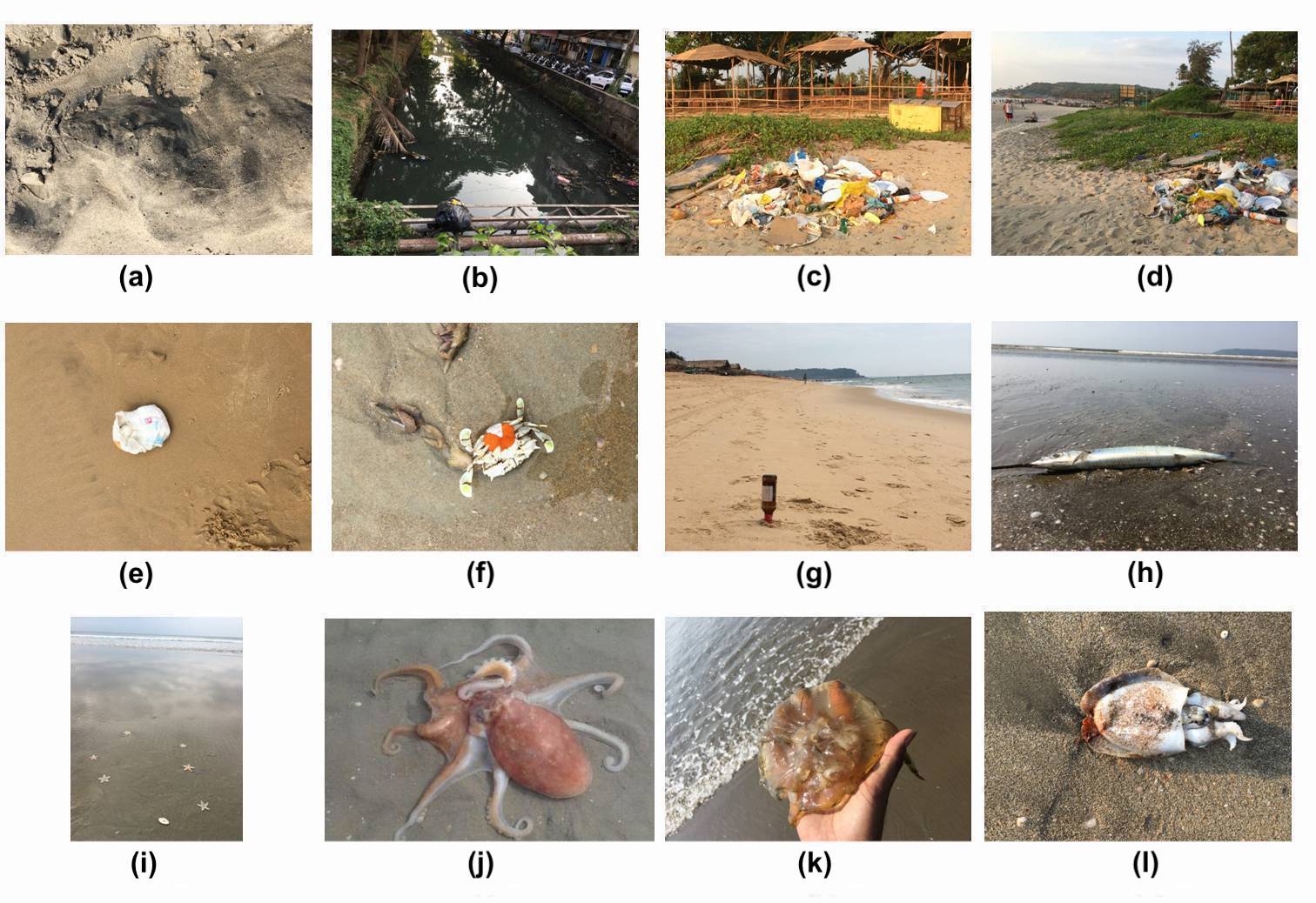
**S Fig. 1.** Impacts of oil pollution at the GA coastline: a) oil pollution; b) a stream of domestic waste dumping into the Arabian Sea at Panjim; c) and d) plastic pollution at beaches; e) used diapers on beaches; f) dead crab; g) an alcoholic beverage bottle; h) dead fish; i) dead starfish; j) live octopus; k) live jellyfish; and l) dead and rotting squid.


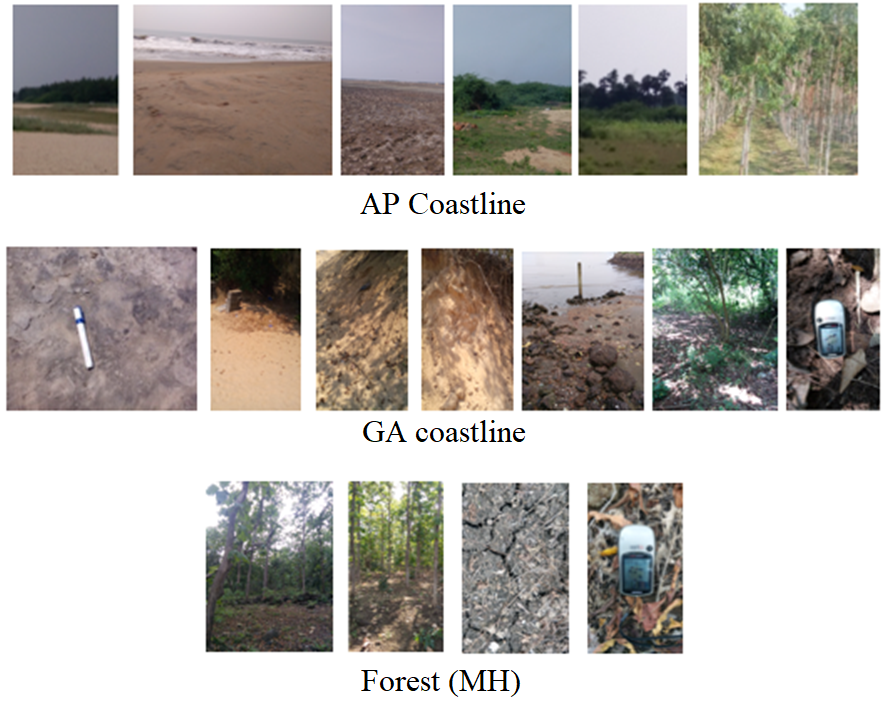


**S Fig. 2.** Sampling sites are located at different geographical locations along the coastlines of AP, GA, and KF. Sampling sites across AP have more vegetation than GA, followed by the forest. Whereas human intervention was minimal in Forest, followed by AP and GA.


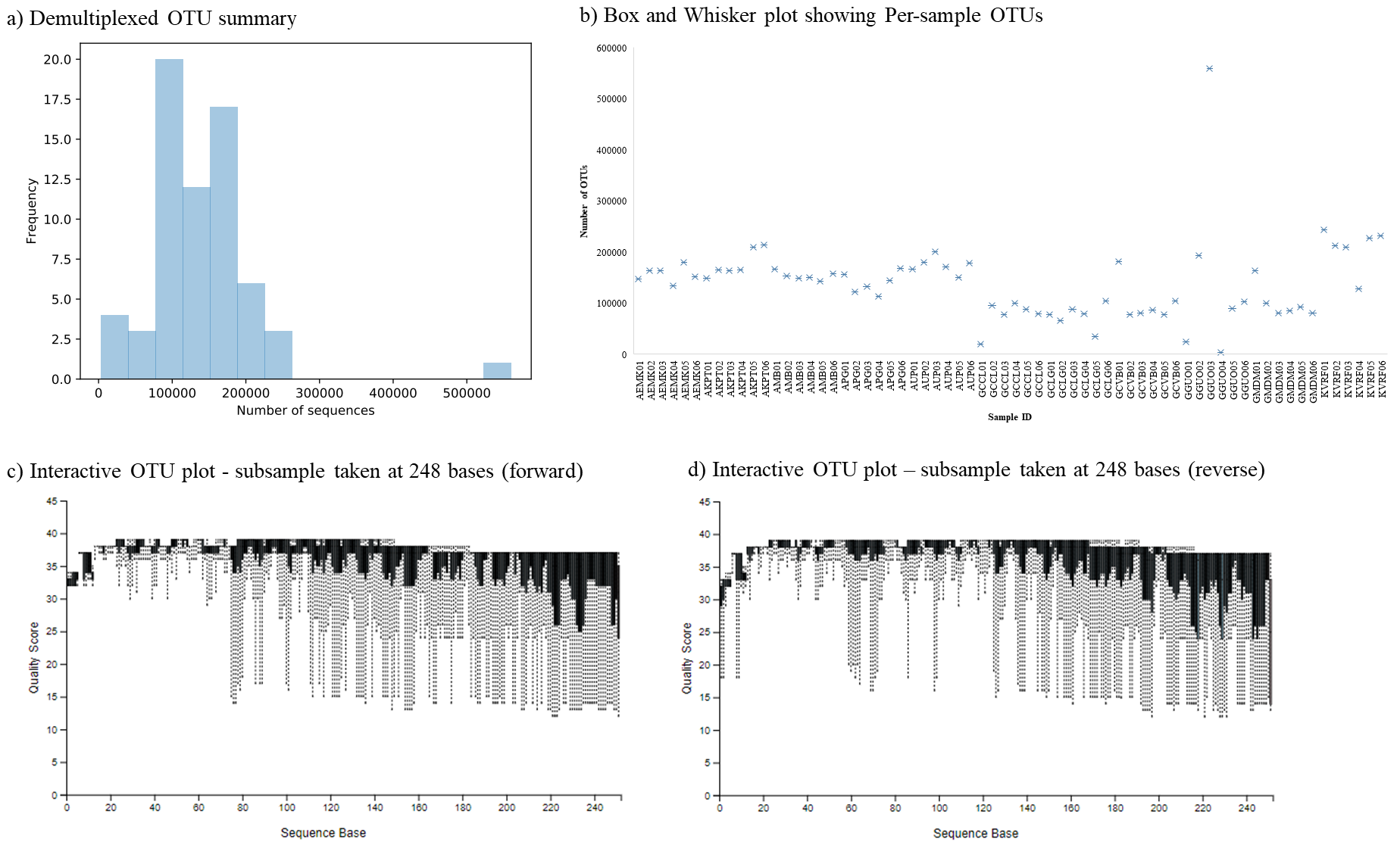


**S Fig. 3.** Feature details: a) Demultiplexing of sequences showing maximum, median, mean, and minimum features; b) Box and Whisker plot highlights number of sequences in each sampling zone; c) and d) Interactive OTU plots at random sampling of 10000 without replacement. Outlier quality scores are not shown in box plots for clarity.


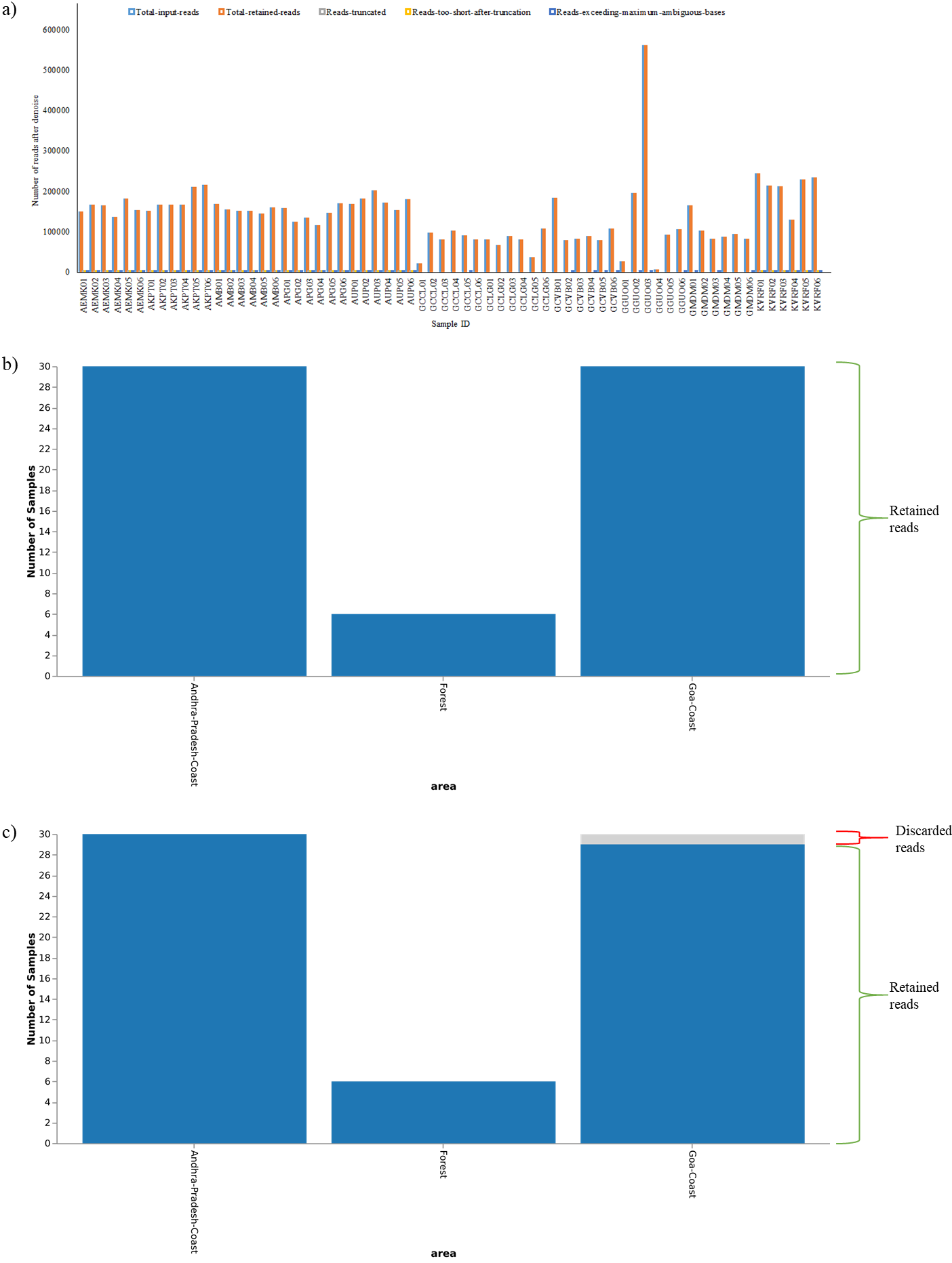


**S Fig. 4.** Number of features retained after denoising (a) at sampling depth 904 (b) and 10000 (b) in each sampling area such as AP, GA, and forest.


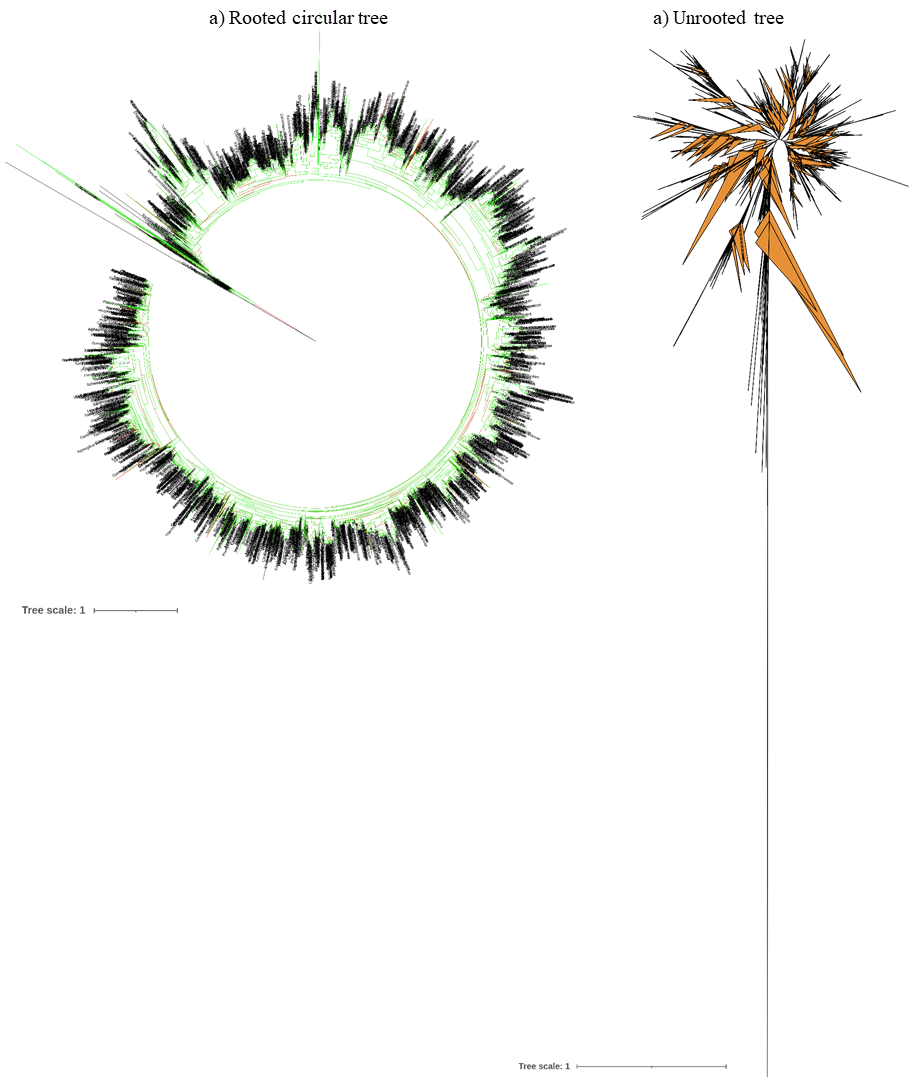


**S Fig. 5.** FastTree: Rooted circular (a) and unrooted tree (b) generated using Interactive Tree of Life v6.


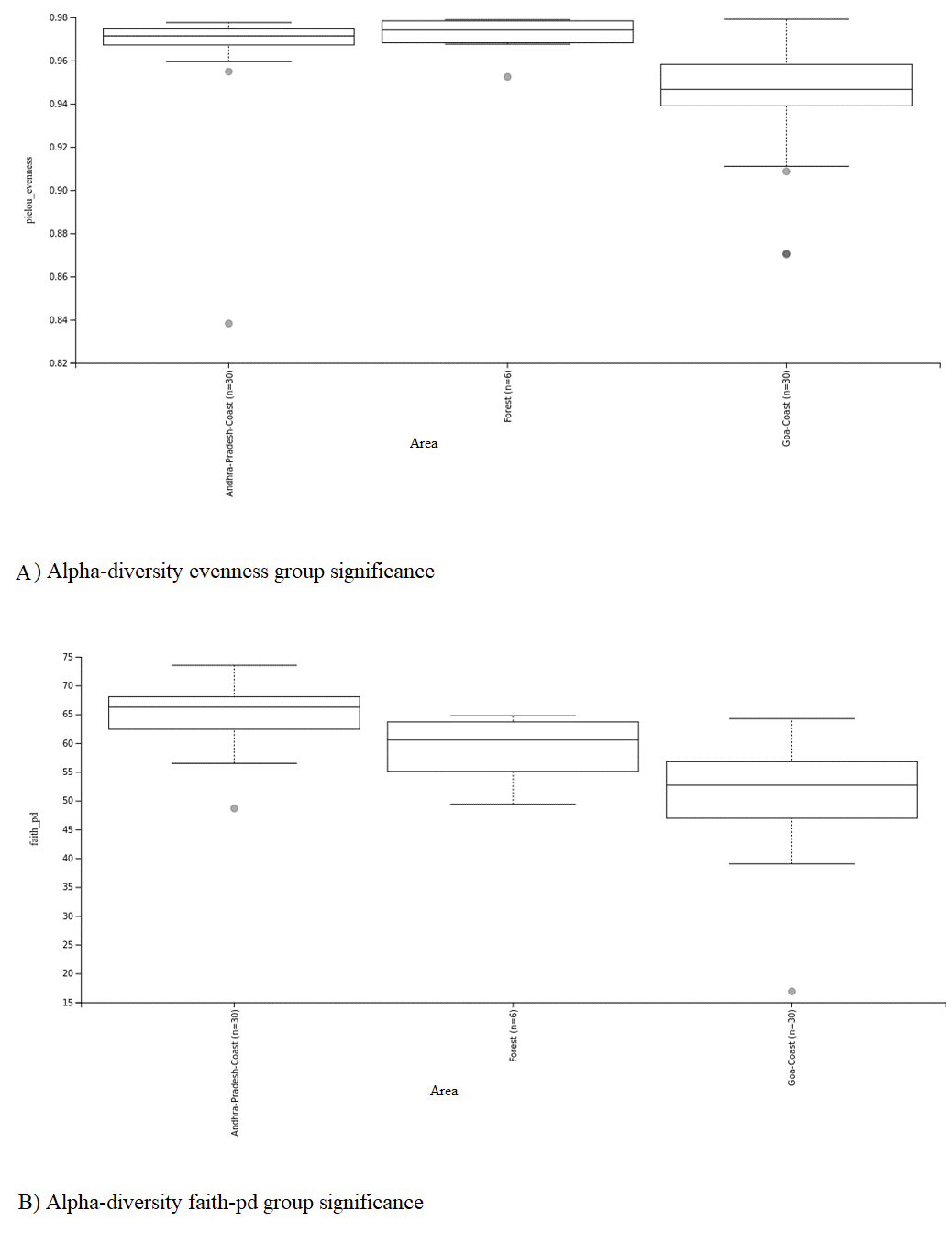


**S Fig. 6.** The alpha diversity of AP, GA and forest - A higher length of the standard bar in evenness (a) and Faith phylogenetic diversity (Faith FD) indicates more diversity within each blox, and vice versa.


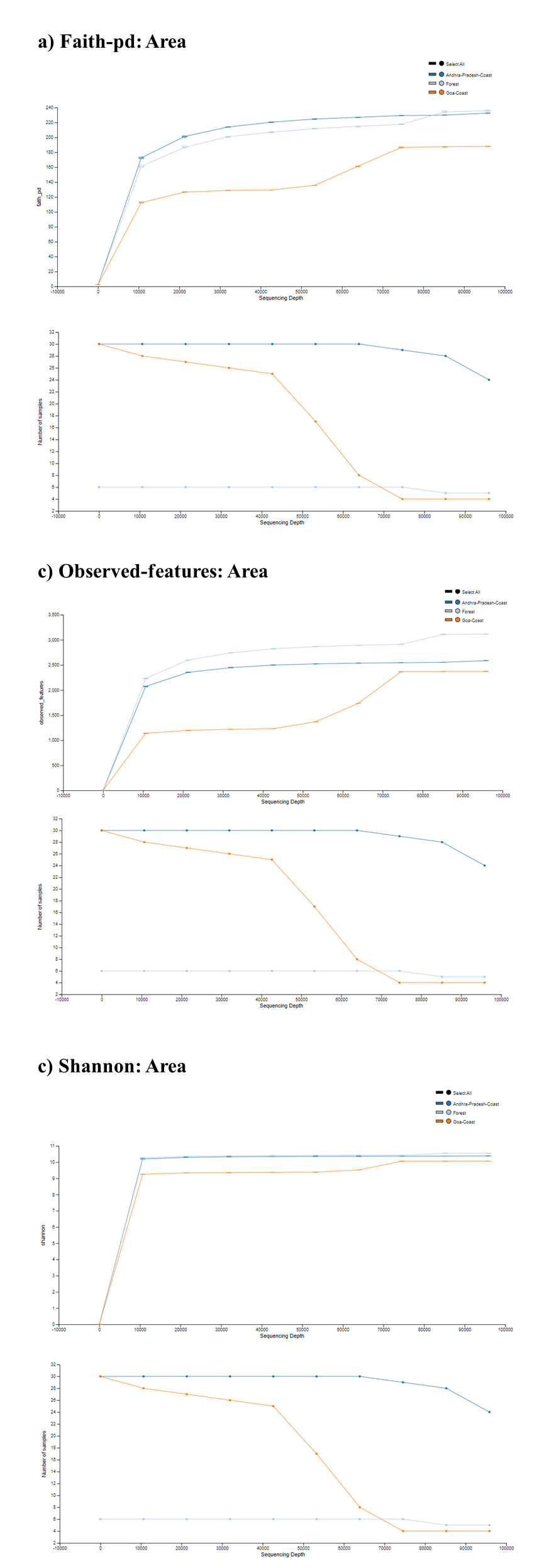
**S Fig. 7.** The alpha diversity rarefaction plots: Cobalt blue represents the AP coast, light blue represents the forest, and orange represents the GA coast. The length of the curve represents how many samples were sequenced. The higher the sequencing depth and the greater the chance of discovering higher diversity, the longer the curve.


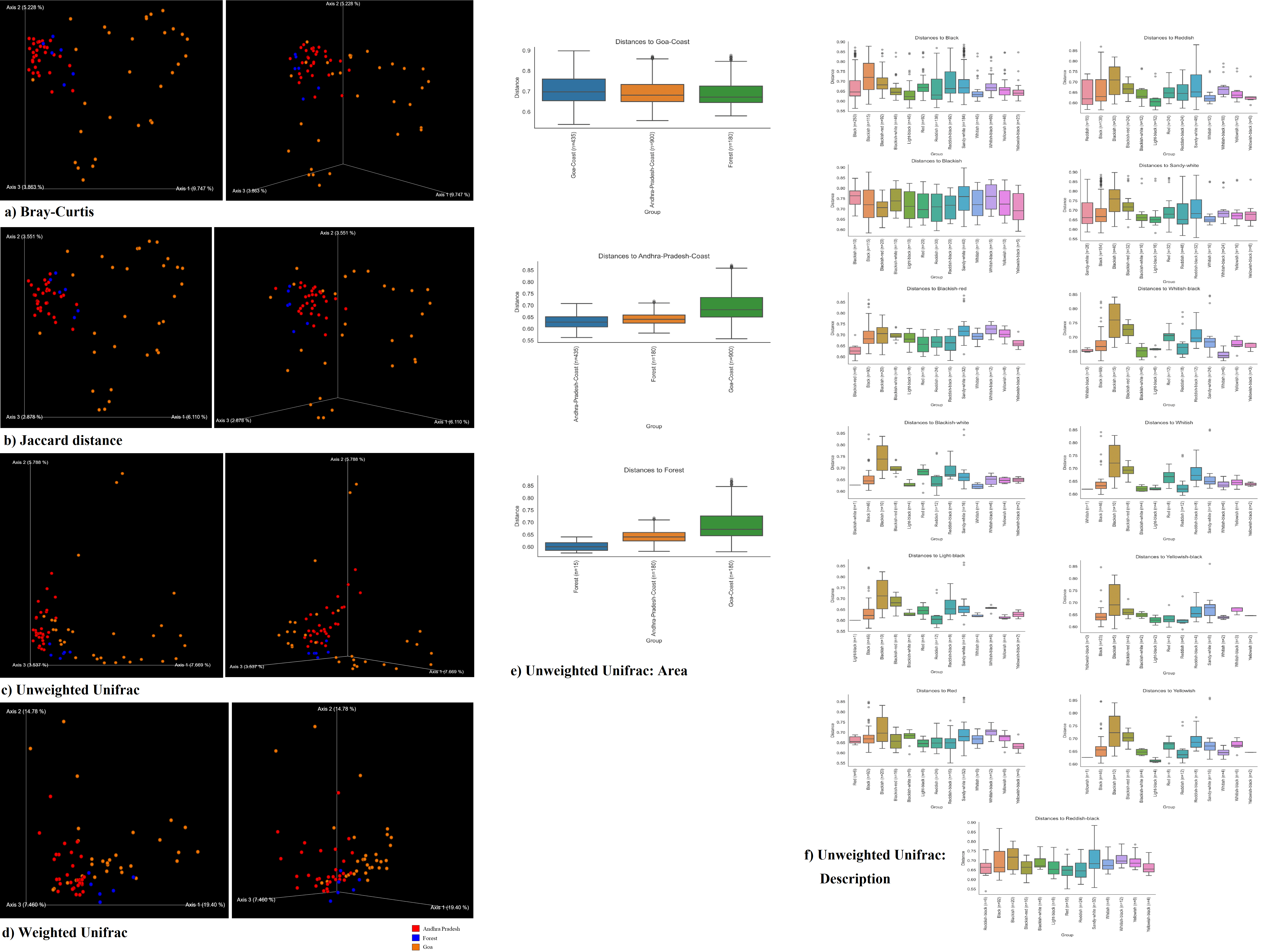


**S Fig. 8.** Beta-diversity: Principal coordinate analyses (PCoAs) were performed through Core-matric analysis. Diversity indices such as a) Bray-Curtis diversity matrices; b) Jaccard-distance; c) Unweighted Unifrac; and d) Weighted Unifrac were measured to state the diversity of the microbial community between samples. Separate analysis using unweighted unifrac was performed to understand the difference between the microbial composition of samples from the (e) area and those based on the (f) description of samples.


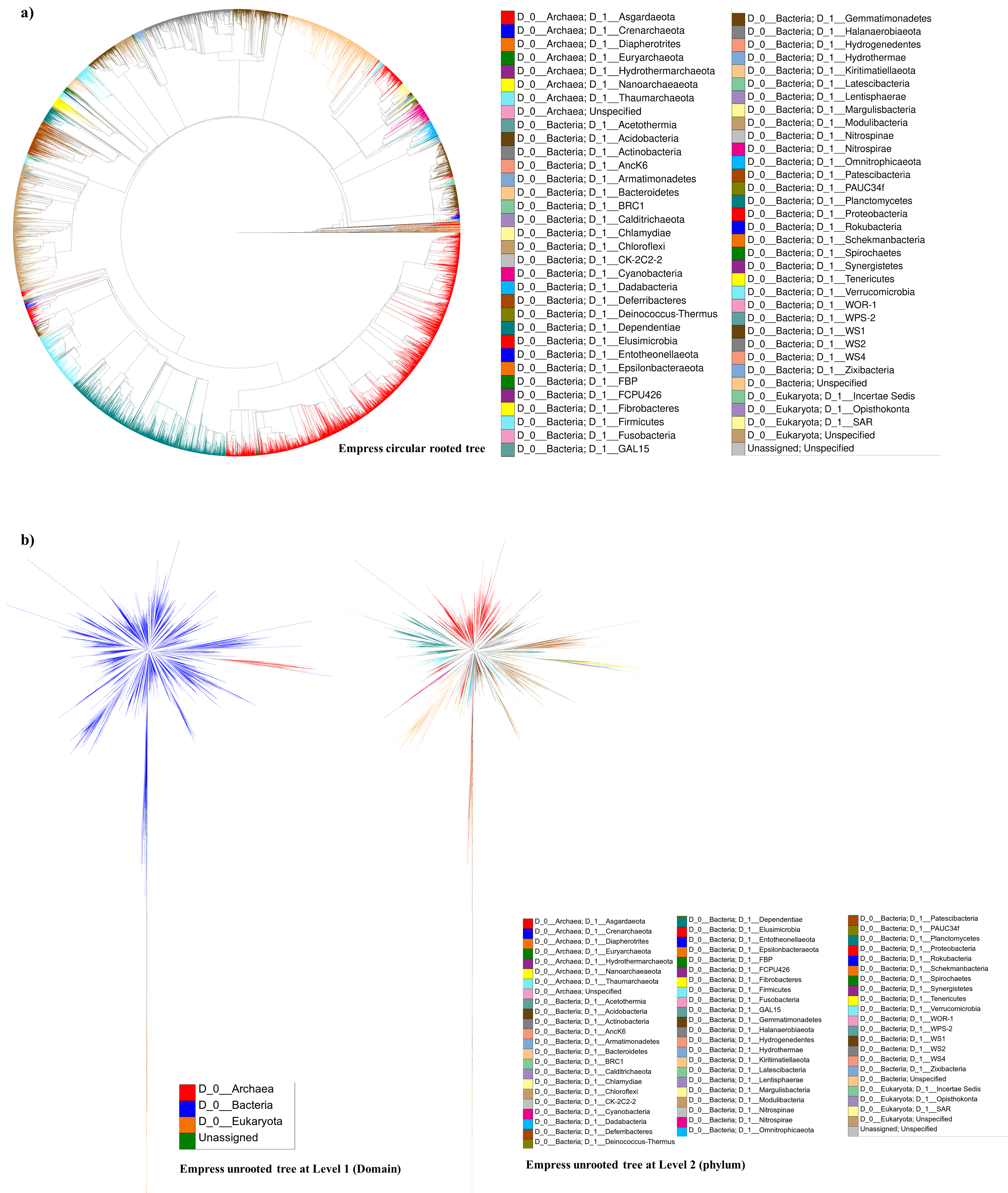


**S Fig. 9.** Empress circular (a) and unrooted tree (b) represents the hierarchical relationships between features in a dataset of AP, GA, and forest. A type of "feature" can be viewed at the plylum level as amplicon sequence variants (ASVs) or operational taxonomic units (OTUs).


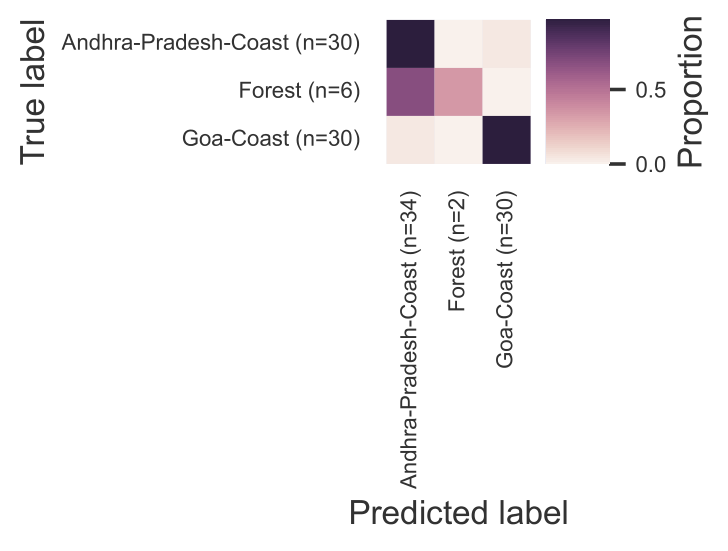


**S Fig. 10.** Random forest regression analysis using a supervised machine learning model to predict microbial composition and create normalized confusion matrices to understand the distribution of diversity across samples.
